# Global, regional, and national quality of care index of cervical and ovarian cancer: a systematic analysis for the global burden of disease study 1990–2019

**DOI:** 10.1186/s12905-024-02884-9

**Published:** 2024-01-25

**Authors:** Mohammadreza Azangou-Khyavy, Erfan Ghasemi, Negar Rezaei, Javad Khanali, Ali-Asghar Kolahi, Mohammad-Reza Malekpour, Mahsa Heidari‐Foroozan, Maryam Nasserinejad, Esmaeil Mohammadi, Mohsen Abbasi-Kangevari, Seyyed-Hadi Ghamari, Narges Ebrahimi, Sogol Koolaji, Mina Khosravifar, Sahar Mohammadi Fateh, Bagher Larijani, Farshad Farzadfar

**Affiliations:** 1 Non-Communicable Diseases Research Center, Endocrinology and Metabolism Population Sciences Institute, Tehran University of Medical Sciences, No. 10, Al-E-Ahmad and Chamran Highway Intersection, Tehran, Iran; 2 Social Determinants of Health Research Center, Shahid Beheshti University of Medical Sciences, Tehran, Iran; 3 Student Research Committee, School of Medicine, Shahid Beheshti University of Medical Sciences, Tehran, Iran; 4 Endocrinology and Metabolism Research Center, Endocrinology and Metabolism Clinical Sciences Institute, Tehran University of Medical Sciences, Tehran, Iran

**Keywords:** Cervical cancer, Ovarian cancer, Healthcare quality improvement, Primary component analysis, Global burden of disease, Socio-demographic index

## Abstract

**Background and objective:**

Cervical cancer is the most preventable and ovarian cancer is the most lethal gynecological cancer. However, in the world, there are disparities in health care performances resulting in differences in the burden of these cancers. The objective of this study was to compare the health-system quality of care and inequities for these cancers using the Quality of Care Index (QCI).

**Material and methods:**

The 1990–2019 data of the Global Burden of Disease (GBD) was analyzed to extract rates of incidence, prevalence, mortality, Disability-Adjusted Life Years (DALYs), Years of Life Lost (YLL), and Years of healthy life lost due to disability (YLD) of cervical and ovarian cancer. Four indices were developed as a proxy for the quality of care using the above-mentioned rates. Thereafter, a Principal Components Analysis (PCA) was applied to construct the Quality of Care Index (QCI) as a summary measure of the developed indices.

**Results:**

The incidence of cervical cancer decreased from 1990 to 2019, whereas the incidence of ovarian cancer increased between these years. However, the mortality rate of both cancers decreased in this interval. The global age-standardized QCI for cervical cancer and ovarian cancer were 43.1 and 48.5 in 1990 and increased to 58.5 and 58.4 in 2019, respectively. QCI for cervical cancer and ovarian cancer generally decreased with aging, and different age groups had inequitable QCIs. Higher-income countries generally had higher QCIs for both cancers, but exceptions were also observed.

**Conclusions:**

Uncovering disparities in cervical and ovarian cancer care across locations, Socio-Demographic Index levels, and age groups necessitate urgent improvements in healthcare systems for equitable care. These findings underscore the need for targeted interventions and prompt future research to explore root causes and effective strategies for narrowing these gaps.

**Supplementary Information:**

The online version contains supplementary material available at 10.1186/s12905-024-02884-9.

## Background

Cervical and ovarian cancers are two types of gynecological malignancies affecting the female reproductive system. Cervical cancer was diagnosed in 601,000 women and caused 260,000 deaths across the globe in 2017 [1]. Worldwide, 1 in 65 women in different age groups dealt with cervical cancer. Ovarian cancer affected 286,000 women and caused 176,000 deaths globally in 2017 [1]. Although regional variability existed, the global all-ages incidence and prevalence numbers of both cervical and ovarian cancer increased from 1990 to 2017 [2].

Cervical cancer is the most preventable and ovarian cancer is the most lethal gynecological cancer [3]. However, in the world, there are disparities in health care performances resulting in differences in the incidence, prevalence, and mortality of these cancers [3–5]. These disparities are caused by systemic factors including different healthcare delivery systems, provider factors such as diverse clinical decisions, and patient factors like barriers to care [3]. Therefore, it is of significant importance to quantify the health care performance and identify the gaps in the need for improvement [6].

Although incidence, prevalence, mortality, disability-adjusted life years (DALYs), years of life lost (YLLs), and years lived with disability (YLDs) of cervical and ovarian cancer could be utilized to provide insights into the health care performances worldwide, none of them is single-handedly sufficient in benchmarking the performance of various health care systems. In this regard, the Healthcare Access and Quality Index (HAQ) has previously been introduced based on the mortality-to-incidence ratios by the Global Burden of Disease (GBD) Healthcare Access and Quality Collaborators [7]. The latter is susceptible to shortcomings as only considering staying alive as a measure of health care access and quality. Previous studies have investigated the quality of care for different diseases using a different and more comprehensive index, the Quality of Care Index (QCI) [8–13]. However, the global quality of care for cervical and ovarian cancer is yet to be studied. In this study, we will compare the health-system quality of care and inequities for cervical and ovarian cancer among various age groups in different nations and regions using the QCI.

## Materials and methods

### Overview

In this study, we analyzed 1990-2019 data of the GBD to extract crude and age-standardized rates of incidence, prevalence, mortality, DALY, YLL, and YLD attributable to cervical and ovarian cancers. The aforementioned six indices were combined, and four secondary indices, namely, mortality to incidence ratio, DALYs to prevalence ratio, prevalence to incidence ratio, and YLLs to YLDs ratio were acquired, all of which partly indicated the quality of care. The four secondary indices were then combined using Primary Component Analysis (PCA) to form a single tertiary index termed QCI. All of the detailed steps of data acquisition, curation, and analysis for generating QCI with their related R programming codes are available from the QCI protocol which has been published previously [14]. The QCI provides an overall re-scaled score of 0-100 reflecting the health care quality at various locations, among different SDI levels and age groups, between 1990 and 2019.

### Data source

The data of the study were extracted from GBD 2019, which was conducted by the Institute of Health Metrics and Evaluation (IHME). GBD 2019 included 204 countries, seven super-regions, and 21 regions from 1990 to 2019 and a systematic analysis of 369 diseases and injuries, and 87 risk factors [15, 16]. The cervical and ovarian cancer data were extracted from GBD 2019 with the GBD codes B.1.15 and B.1.17 respectively [17]. The classifications and sub-categories of cervical and ovarian cancers used in this study are based on the International Statistical Classification of Diseases and Related Health Problems 10th Revision, World Health Organization version (ICD-10) codes available from James SL et al. and Roth GA et al. studies. [2, 18]. For categorizing countries based on development status, the GBD Socio-Demographic Index (SDI) was used [19].

### Statistical analysis

#### Quality of care index

As stated above, the secondary indices were defined as follows:

$$\mathrm{Mortality \,to \,incidence \,ratio}=\frac{\#\;Mortality}{\#\;Incidence}$$

$$\mathrm{DALY \,to \,prevalence \,ratio}=\frac{\#\;\mathrm{DALY }}{\#\;{\text{Prevalence}}}$$

$$\mathrm{Prevalence \,to \,incidence \,ratio}=\frac{\#\;\mathrm{Prevalence }}{\#\;Incidence}$$

$$\mathrm{YLL \,to \,YLD \,ratio}=\frac{\#\;\mathrm{YLL }}{\#\;{\text{YLD}}}$$

#### Principal components analysis

In this study, PCA was performed to convert the four secondary indices, indicating the quality of care, into a single tertiary index [20], which was termed QCI. The QCI ranged from 0 to 100, in which, 100 indicated the best quality of care. PCA was performed using R software version 3.5.2. To make the data searchable and retrievable, QCI for cervical and ovarian cancer was categorized into five levels in 2019 based on 20 percentiles, where Level 1 indicated the highest index, and Level 5 the lowest. Categorizing the QCI data into five levels has the added benefit of giving the big picture of the quality of care at various locations at the same level despite the differences in details. The QCI data based on locations for cervical and ovarian cancers is presented in Supplementary tables 1 and 3. The colors in these tables indicate the QCI level in 2019.

#### Data validation

To evaluate the validity of the QCI and the data, the correlation between the QCI and the Healthcare Access and Quality (HAQ) index [7, 21] was determined by applying a mixed-effect model. In this model, QCI was a dependent variable, and inpatient and outpatient health care utilization, cervical and ovarian cancer, mortality, prevalence, and attribute mortality to all risk factors were independent variables. Countries were considered as random effects. The correlations between the HAQ Indices and the predicted values for cervical and ovarian cancer were 0.81 and 0.64 respectively, which were acceptable.

The statistical analyses were conducted using R statistical packages v3.4.3 (http://www.r-project.org, RRID: SCR_001905). Data visualizations were carried out using Python programming language (Python Language Reference, version 3.6. Available at www.python.org) via Altair version 4.1, an open-source Python library in addition to the R statistical packages v3.4.3 (http://www.r-project.org, RRID: SCR_001905).

## Results

### Cervical cancer

#### Prevalence, incidence, and mortality

In 1990 the global age-standardized prevalence of cervical cancer was 66.2 (60 to 76.5) per 100,000, which increased to 69.1 (58.3 to 77.1) in 2019. However, not all regions followed the global trend of cervical cancer prevalence. E.g., the prevalence decreased -7.3% (-17 to 4.2) in European, and -10.8% (-27.2 to 15.1) in Southeast Asia region (Supplementary Table 1).

The global incidence of cervical cancer was 14.9 (13.4 to 17.5) per 100,000 in 1990, which decreased -10.4% (-23.5 to 0.9) to 13.4 (11.4 to 15) per 100,000 in 2019. The same decreasing trend was observable in all world regions (i.e., based on WHO regions) except for the Western Pacific region which increased by 13.8% (-41 to 50.6). The global mortality rate of cervical cancer - 23.2% (-34.7 to - 12.3) decreased from 8.5 (7.6 to 10.1) per 100,000 in 1990 to 6.5 (5.5 to 7.3) per 100,000 in 2019. The highest mortality rate of cervical cancer in both 1990 and 2019 was in the African region countries, while the lowest mortality rate was observed in the Eastern Mediterranean countries (Supplementary Table 1).

#### Quality of care index

##### Global, regional, and country level

**Fig. 1:**
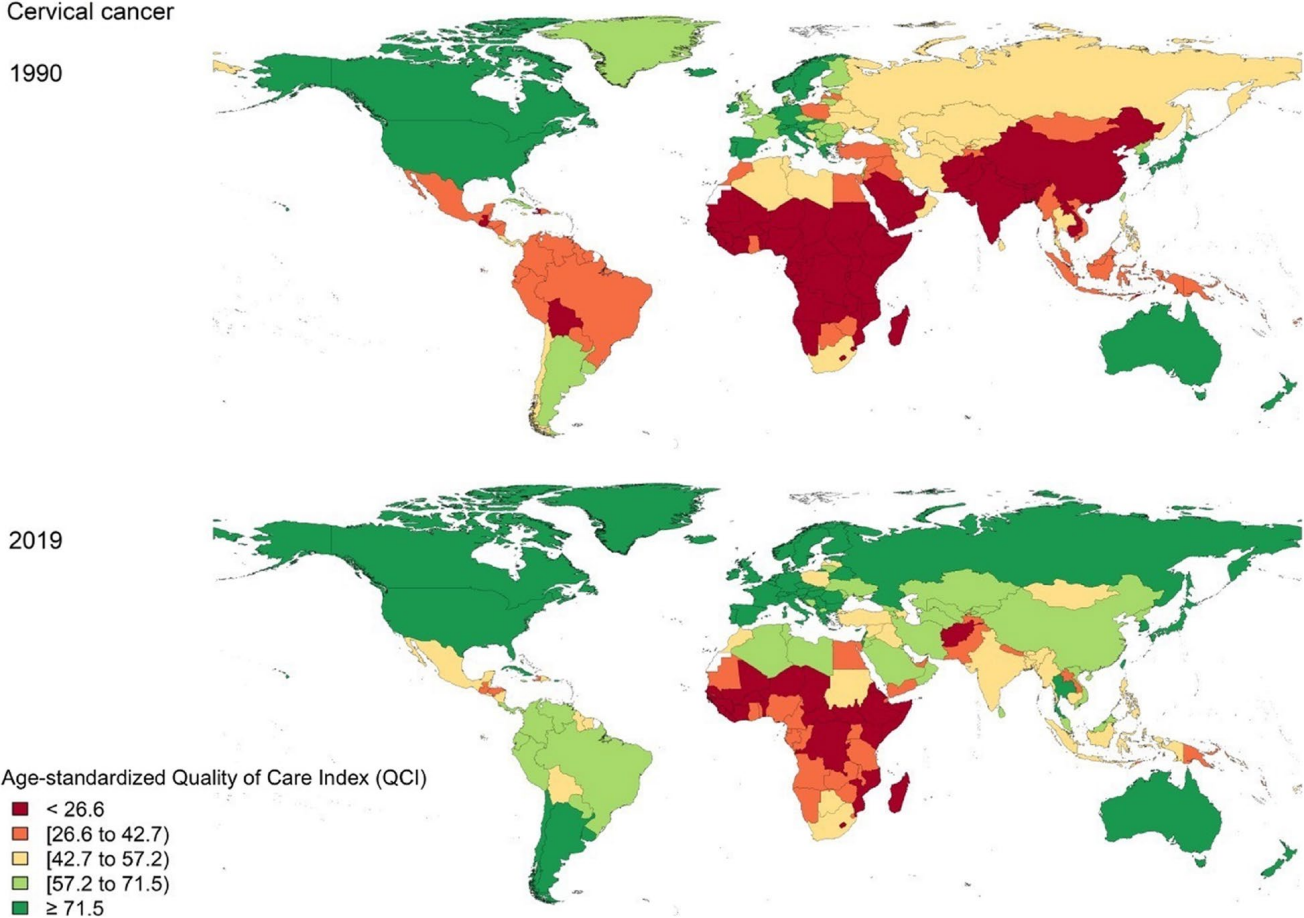
Quality of Care Index (QCI) for cervical cancer by country in 1990 and 2019

Divergent patterns of QCI were observable across the globe for cervical cancer from 1990 to 2019. The global age-standardized QCI was 43.1 in 1990, which has a 35.8% increase to 58.5 in 2019. Among WHO regions, the highest quality of care for cervical cancer was 62.7 (QCI level 4) in 1990 and 76.6 (QCI level 5) in 2019, both of which attributed to the European region. On the other hand, the African region had the lowest quality of care for cervical cancer in both years (i.e., QCI levels 1 and 2) (Supplementary Table 2). All of the countries and territories quality of care for cervical cancer has variably increased from 1990 to 2019 except for Zimbabwe (-9.2%), Tajikistan (-3.6%), and Somalia (-1.6%) (Fig. 1). The top five countries with the highest QCI (QCI level 5) for cervical cancer in 2019 were Canada (99.4), Australia (95.8), Japan (95.4), Spain (93.2), and Slovenia (90.8). Somalia (7.6), Central African Republic (7.7), South Sudan (14.3), Chad (15.9), and Niger (19.7) had the lowest quality of care for cervical cancer (QCI level 1) (Fig. 1 and Supplementary Table 2).

##### World Bank income and SDI levels

**Fig. 2:**
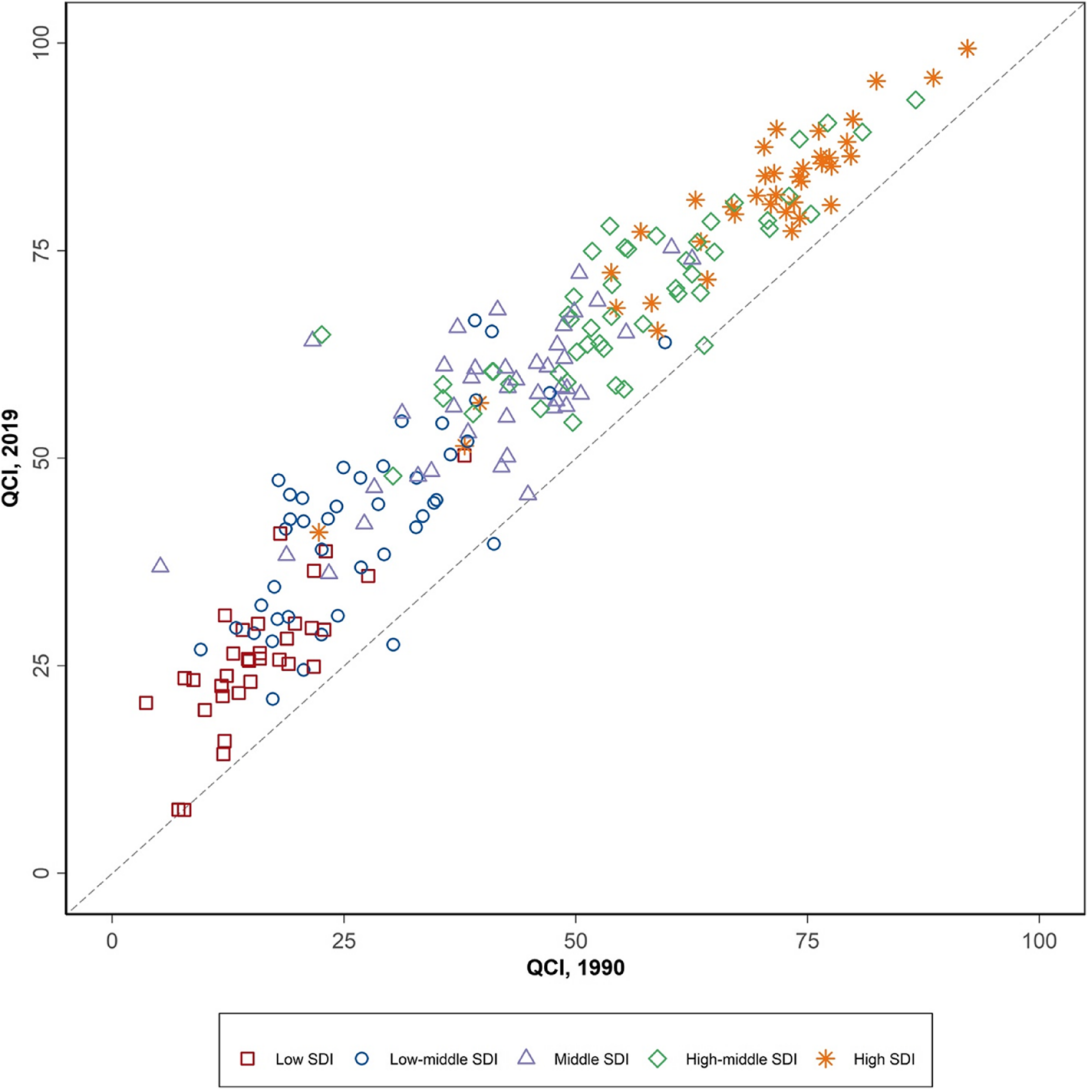
Quality of Care Index (QCI) for cervical cancer by Socio-Demographic Index (SDI) score of countries in 1990 and 2019

Age-standardized QCI for cervical cancer in World Bank high-income countries increased 14.4% from 73.7 to 84.3 from 1990 to 2019. The QCI of World Bank low-income countries increased by 54.4% from 18.1 in 1990 to 27.9 in 2019 (Supplementary Table 2). In both years, income level was positively correlated with the quality of care for cervical cancer. The correlation between the quality of care for cervical cancer and the SDI levels was exactly similar (Fig. 2 and Supplementary Table 2). Although the quality of care for cervical cancer increased in almost all countries in all SDI levels since 1990 (Fig. 2), low SDI countries had the highest increase rate for cervical cancer QCI (i.e., 87.8%).

##### Age Pattern

Globally, QCI for cervical cancer decreased with aging, and different age groups had inequitable QCIs. Moreover, from 1990 to 2019, the cervical cancer QCI increased in all age groups to various extents. The highest QCI was observed in the 30 to 34 age group in both 1990 and 2019 (i.e., 52.7 and 67.5). On the other hand, the lowest QCI was in the over 80 age group in 2019 (i.e., 40.6) and in the 75 to 59 age group in 1990 (i.e., 34) (Supplementary Table 3).

**Fig. 3:**
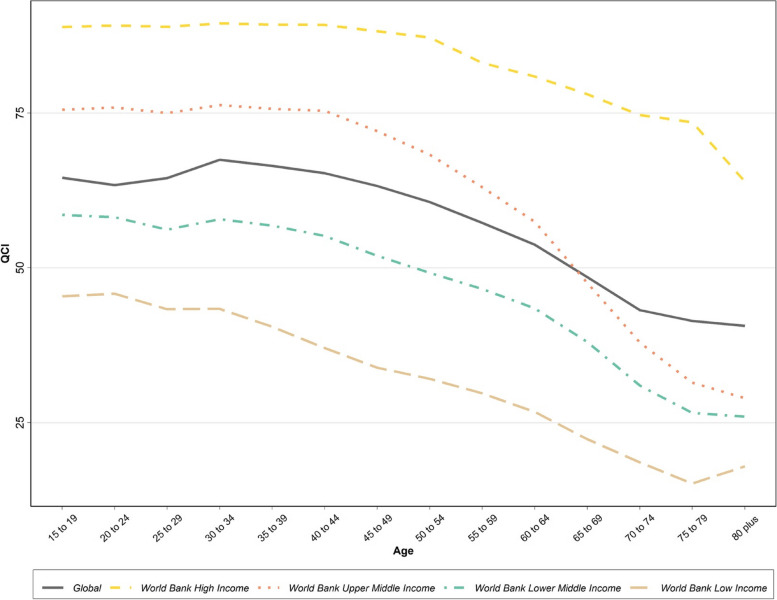
Age pattern for Quality of Care Index (QCI) for cervical cancer based on World Bank income levels in 2019

At the country level, categorized by income level, even though a similar decreasing trend was observed among all income levels, the QCI was higher among all age groups in high-income countries, and for each age group, the QCI was positively correlated with the income level **(**Fig. 3).

### Ovarian cancer

#### Prevalence, incidence, and mortality

The global age-standardized prevalence of ovarian cancer increased from 24.7 (22.8 to 28.1) per 100,000 in 1990 to 28.6 (25.2 to 32.1) in 2019. However, it decreased -3.3% (-13.9 to 16.6) in Europe, and -6.4% (-18.7 to 9.9) in the region of the Americas (Supplementary Table 4).

The global incidence of ovarian cancer was 6.5 (6 to 7.3) per 100,000 in 1990 and increased to 6.9 (6.1 to 7.7) per 100,000 in 2019. The age-standardized incidence increased in all world regions except for the European region which decreased -8.4% (-17.8 to 8.5) and the region of the Americas which decreased -10.5% (-21.8 to 4.4). The global mortality rate of ovarian cancer remained approximately stagnant from 1990 to 2019 [i.e., 4.6 (4.2 to 5.2) Vs. 4.6 (4 to 5)]. The highest mortality rate of ovarian cancer in both 1990 and 2019 was in the European region countries, while the lowest mortality rate was observed in the Western Pacific region countries (Supplementary Table 4).

#### Quality of care index

##### Global, regional, and country level

**Fig. 4:**
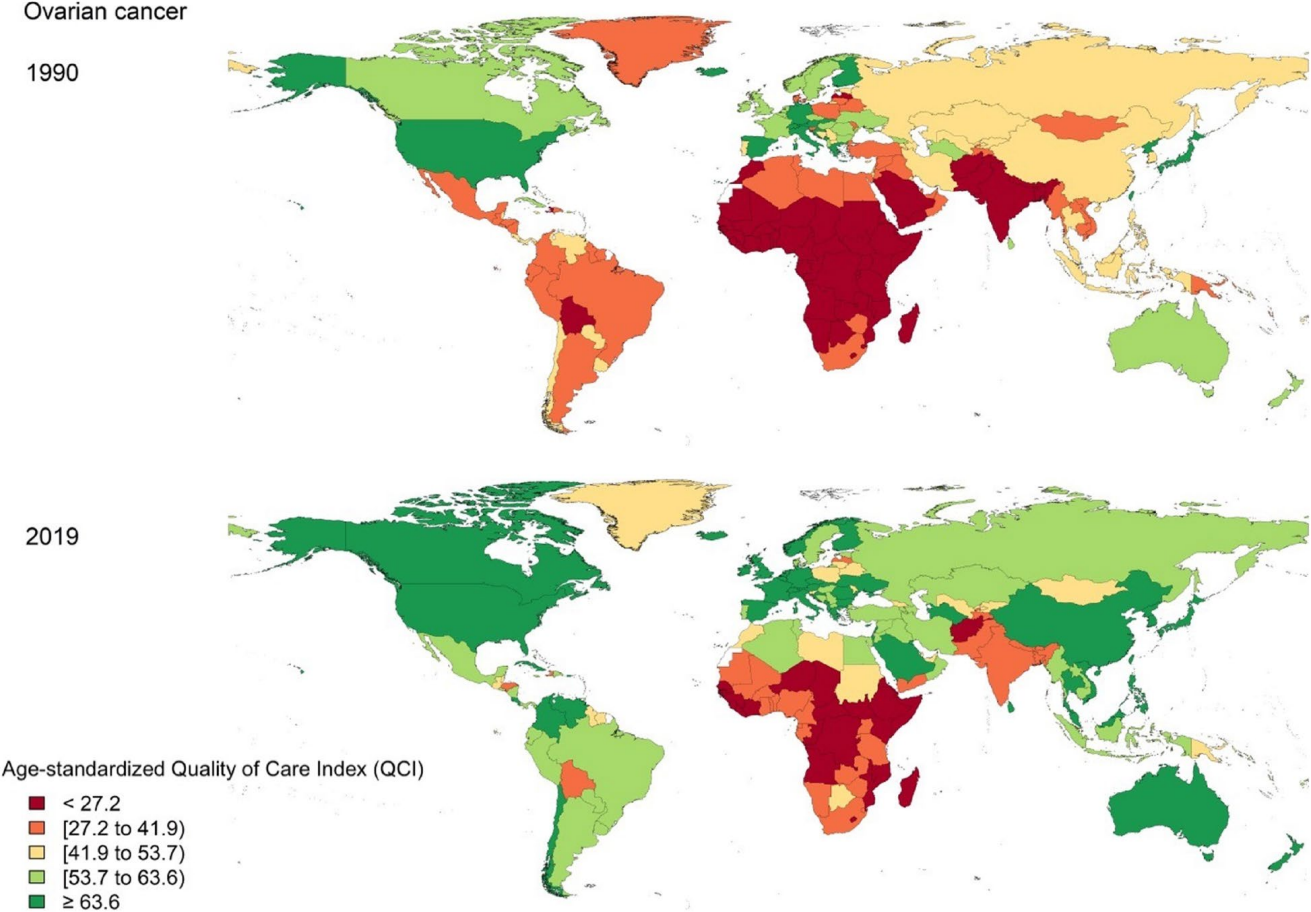
Quality of Care Index (QCI)for ovarian cancer by country in 1990 and 2019

QCI for ovarian cancer differed across the globe (Fig. 4). The global age-standardized QCI was 48.5 in 1990, which has a 20.5% increase to 58.4 in 2019. Among WHO regions, the highest quality of care for ovarian cancer was in the European region in 1990 (i.e., 58.4) and in the Western Pacific region in 2019 (72.6). On the other hand, the African region had the lowest quality of care for ovarian cancer in both years (i.e., QCI levels 1 and 2) (Supplementary Table 5). All of the countries and territories quality of care for ovarian cancer has variably increased from 1990 to 2019 except for countries including Georgia (-9.3%), Kenya (-7.2%), and Dominica (-1.9%) (Fig. 4). The top five countries with the highest QCI (QCI level 5) for ovarian cancer in 2019 were Taiwan (100), Spain (92), Japan (91.4), Croatia (87), and the Republic of Korea (86.1). Somalia (10.1), Central African Republic (12.3), South Sudan (15.4), Eritrea (19.2), and Kenya (20) had the lowest quality of care for ovarian cancer (QCI level 1) (Fig. 4 and Supplementary Table 5).

##### World Bank income and SDI levels

**Fig. 5:**
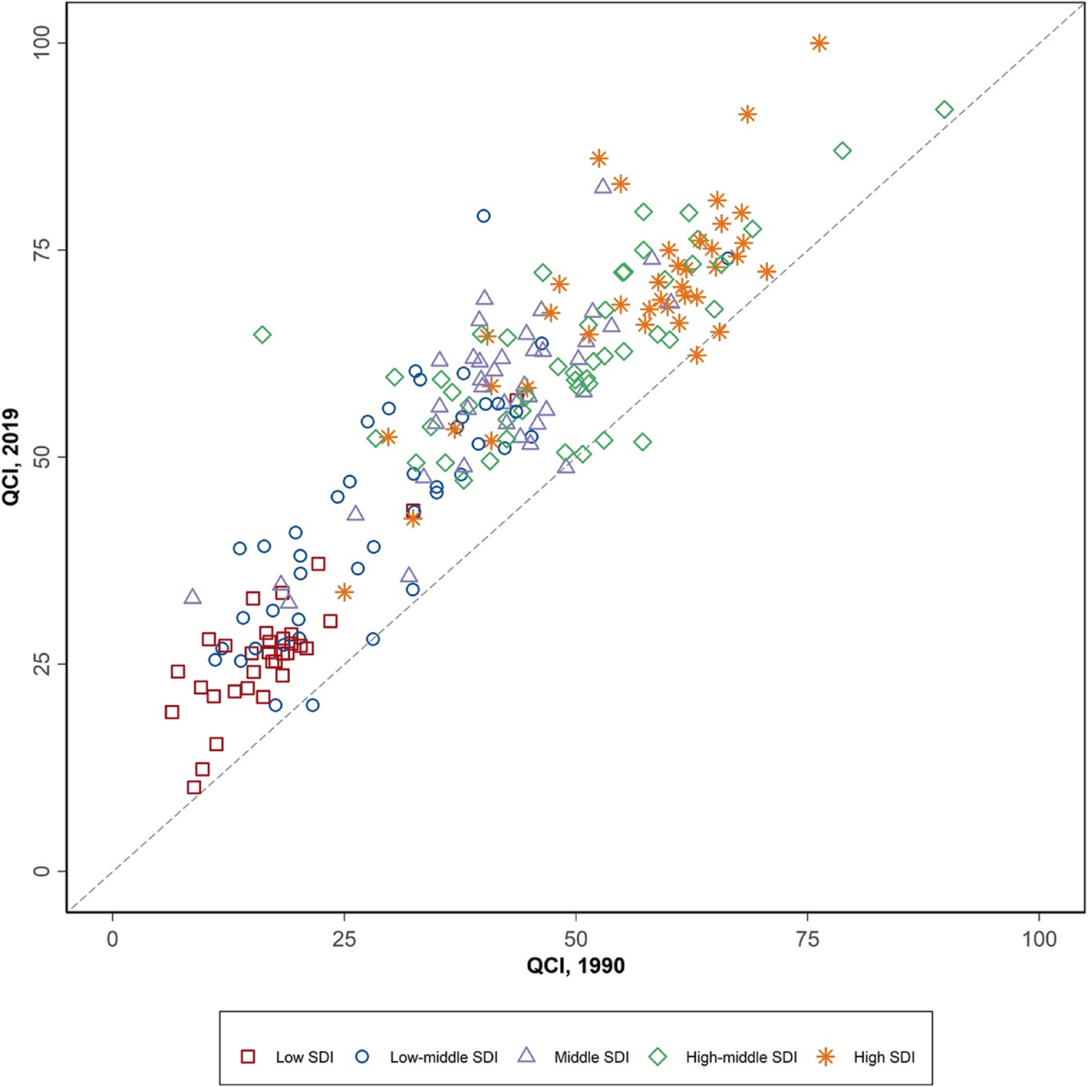
Quality of Care Index (QCI) for ovarian cancer by Socio-Demographic Index (SDI) score of countries in 1990 and 2019

World Bank high-income countries had the highest age-standardized QCI for ovarian cancer in 1990 and 2019. However, the QCI of World Bank low-income countries increased by 46.2% during this period (Supplementary Table 5). In both years, income level was positively correlated with the quality of care for ovarian cancer. SDI level had a similar correlation as well (Fig. 5 and Supplementary Table 5). Although the quality of care for ovarian cancer increased in almost all countries in all SDI levels since 1990 (Fig. 5), low SDI countries had the highest increase rate for ovarian cancer QCI (i.e., 92.9%).

##### Age Pattern

Quality of care for ovarian cancer generally decreases with aging. From 1990 to 2019, the ovarian cancer QCI increased in almost all age groups to various extents. The highest QCI was observed in the 25 to 29 age group in both years (i.e., 61.7 and 71.8). On the other hand, the lowest QCI was in the 75 to 79 age group in both 1990 (i.e., 48.9) and 2019 (i.e., 51.6) (Supplementary Table 6).

**Fig. 6:**
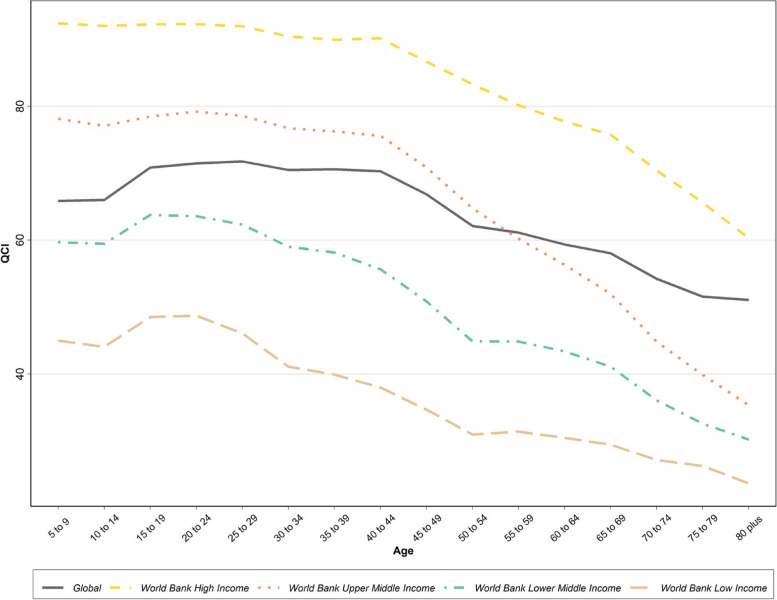
Age pattern for Quality of Care Index (QCI) for ovarian cancer based on World Bank income levels in 2019

It is also worth noting that the QCI was higher among all age groups in high-income countries and for each age group, the quality of care was positively correlated with the income level (Fig. 6).

## Discussion

The global age-standardized QCI for cervical and ovarian cancer increased from 1990 to 2019 in all income levels. However, the gap between the highest and lowest QCI for both cancers in 2019 was higher than in 1990, which might indicate a more inequitable access to and quality of health care in recent years. This is even though low-income countries had the greatest improvements. Across various age groups, we observed a trend where younger individuals typically exhibited higher QCIs. Specifically, within each designated age range, we found a positive correlation between the QCI and income levels. Plus, high-income countries demonstrated higher QCIs in all age groups. Disparities were also observed in QCI among different age groups for ovarian cancer, and generally, QCI decreased as people aged.

In the case of cervical cancer, prevention through systematic vaccination and screening programs plays a critical role in the management of this cancer in health systems. The Human papillomavirus (HPV) infection is the main cause of developing cervical cancer which is preventable by vaccines. Moreover, the treatment of precancerous lesions diagnosed through the screening protocols has shown proper efficacy [22]. Up to one-third of all diagnosed cancers in many low-resource countries is cervical cancer. In contrast, this proportion is less than 10% in many high-resource countries [23]. Therefore, in high-income countries with sufficient resources for these programs, the incidence and mortality of cervical cancer have more than halved in the last 30 years [1, 24]. Ethiopia, Eritrea, and Equatorial Guinea, all of which as Sub-Saharan African countries had remarkable improvements in quality of care for cervical cancer. This improvement in the QCI in the region might be associated with the GDP increase since the late 1990s. Moreover, the expenditure shares of GDP on the health sector in the Sub-Saharan Africa region have increased [25]. Another example of the support of these programs' effect on the quality of care for cervical cancer is Japan. Japan reached the second rank of QCI for cervical cancer as a high SDI country with widespread screening and vaccination programs [26, 27]. It is also evident that low-resource countries have been slow in implementing HPV vaccination programs [28]. Although there have been increasing controversies concerning the effectiveness of these programs [29, 30], even though cervical cancer is uncommon, the United States is another high-income country possessing systemic screening and vaccination programs resulting in declining incidence and mortality rates of cervical cancer [31]. Besides, it seems evident that cervical cancer screening programs have been a cost-effective measure for the optimum prevention of cervical cancer in Australia and Finland [32, 33]. It is also worth noting that different treatment approaches towards cervical cancer have been updated in the means of diagnostics and staging, imaging, surgical techniques, and targeted therapies [34]. Naturally, these items’ accessibility and affordability are also affected by the socioeconomic status of different nations consequently affecting the quality of care for cervical cancer.

Furthermore, in the Central Asian countries relatively high incidence of cervical cancer is also challenged by the income level in those countries [35]. On the other hand, in 2019, countries with the same SDI levels spread across different QCI levels for both cervical and ovarian cancers (Figs. 2 and 5), indicating that the developmental state was not the only predicting factor in the quality of care of these cancers. Hence, policy actions and focused non-financial investments including increasing public awareness in seeking health care services might be playing roles [36–39]. As an example, comparing the cases of Japan and the United States, both countries share comparable socioeconomic statuses, yet their cervical cancer quality of care differs (95.4 vs. 80.5). This difference can be attributed to various factors, including variations in healthcare infrastructure, screening programs, public awareness, and cultural attitudes towards preventive care. Japan, for instance, has a well-established screening program and a culture that encourages regular health check-ups. In contrast, the United States faces challenges related to healthcare accessibility and disparities, which impact the effectiveness of cervical cancer prevention and early detection efforts [40].

Cervical cancer most commonly develops in women at 30 to 40 ages. However, there is a second incident increase after the age of 70 [41]. This study’s results suggest that the greatest QCI in each income level belongs to younger age groups, which might be primarily due to the better prognosis of cervical cancer in younger ages. Indeed, age seems to be an independent negative prognostic factor. Besides, commonly, the elderly receive more conservative treatments for cervical cancer [42]. Furthermore, pertinent awareness and health-seeking behaviors are more sensible among the younger age groups [36].

However, due to the advances in healthcare and an increase in life expectancy, the elderly population is increasing [43]. Therefore, it is still crucial yet challenging to address the gaps in the cervical cancer healthcare quality that have resulted in lower QCIs in these age groups. Naturally, due to the physiological aging processes and comorbidities, cervical cancer management in the elderly is associated with complications, causing lower quality of care [41].

As mentioned above, vaccination and screening programs are potential tools for managing the cervical cancer burden. The target population of the HPV vaccination is the women before sexual debut. After that, screening programs step in to diagnose more manageable precancerous lesions [44]. However, the efficacy of the vaccination programs is variable and the efficacy of screening programs is discussable due to various influencing factors [29, 30, 45]. Therefore, increasing the quality of care for cervical cancer might be within the realm of possibility through proper decisions on resource allocation and policy making. Accordingly, these decisions have to address the barriers against increasing the efficacy of these programs based on the cost-effectiveness of each program and the specific circumstances of each country. For instance, since the low-and middle-income countries lack the infrastructure required for the cytological screening methods, the World Health Organization has suggested alternative screening methods for these countries such as visual inspection with acetic acid (VIA) [46–48].

In the case of ovarian cancer, despite the advances in screening and treatment methods, it remains the most lethal gynecological cancer. The younger age is usually associated with the early stages of the disease which enhances the survival time of younger patients [49]. In addition, unlike cervical cancer, there is no definite prevention approach for ovarian cancer. However, oral contraceptive usage [50], parity [51], breastfeeding [52], non-steroidal anti-inflammatory drug (NSAID) usage [53], healthy diet [54], physical activity [55], and surgical approaches have been demonstrated to be protective against ovarian cancer [56–58].

Countries with higher resources do not necessarily demonstrate better access to and quality of health care, which might be a reflection of the importance of proper resource management. Furthermore, the increased global QCI for ovarian cancer is consistent with the decreased mortality of this cancer [59, 60]; which might be a result of improvements in disease management.

Similar to cervical cancer, Sub-Saharan African countries including Equatorial Guinea and Ethiopia had the greatest improvements in the QCI for ovarian cancer. Taiwan had the highest QCI level for ovarian cancer in 2019. Taiwan is a high SDI East Asian country that managed to improve ovarian cancer prognosis since 2000. It is believed that this improvement might be associated with upgraded treatment strategies and a better quality of care given by well-trained gynecologic oncologists [61].

As mentioned above, younger patients have better survival times than the elderly. At an income level, there were disparities in QCIs among age groups which might be a reflection of the youngster-weighed resource allocation. However, in a specific age group, the QCI was positively associated with the SDI level.

## Strengths and limitations

The QCI is a more comprehensive index than incidence, prevalence, mortality, DALYs, YLLs, and YLDs alone. Therefore, it reflects a multidimensional estimate of the quality of care among different locations, SDI levels, and age groups. The QCI in this study has also addressed the limitations associated with the HAQ index, including calculations by SDI levels, different age groups, and sexes. Moreover, the HAQ was calculated until 2016 with 5-year intervals, whereas QCI is calculated by year, and 2019 data is also included. However, it is validated through acceptable correlations with the established HAQ indices for cervical and ovarian cancers. Furthermore, the observed QCIs are consistent with other studies justifying our results. However, to calculate this index, we have used the GBD study’s data. Thus, we have experienced the same limitations [18]. For instance, GBD uses estimations when there is scarce data in locations. This lack of precision and other limitations including the quality of data and uncertainties in estimations affect our results as well. Plus, this study does not cover the sub-national QCI levels and possible inequities. In addition, there are some risk factors and protective factors for both cervical and ovarian cancers, some of which were mentioned above. These factors need to be taken into account and adjusted within the QCI analyses. Therefore, future studies with more accurate and detailed data are required to circumvent the acknowledged limitations.

## Conclusion

The global QCI for both cervical and ovarian cancer increased from 1990 to 2019. However, there were disparities at various locations, among different SDI levels, and age groups. The miscellaneous QCIs among countries imply the need to acknowledge the imperative to improve health care systems to reach the aim of equity in the quality of health care. Moreover, evidence-based information can enhance the awareness and health-seeking behaviors among women, and consequently, increase the quality of care. According to our results, neither the age nor the SDI level was sufficient to predict the quality of care for cervical and ovarian cancer at a specific location. Therefore, there have to be other affecting factors that need to be discovered. To accomplish this objective, the reasons behind the QCIs of the pioneer and the terminal countries need to be outlined. Thereafter, pioneer countries could be used as a model for subsequent countries, and therefore, less inequitable quality of care for cervical and ovarian cancer would be within the realm of possibility.

### Supplementary Information

**Additional file 1: Supplementary Table 1.** All ages and age-standardized burden of cervical cancer from 1990 to 2019 in different locations.

**Additional file 2: Supplementary Table 2.** The QCI for cervical cancer from 1990 to 2019 in different locations.

**Additional file 3: Supplementary Table 3.** The QCI for cervical cancer from 1990 to 2019 among different age groups.

**Additional file 4: Supplementary Table 4.** All ages and age-standardized burden of ovarian cancer from 1990 to 2019 in different locations.

**Additional file 5: Supplementary Table 5.** The QCI for ovarian cancer from 1990 to 2019 in different locations.

**Additional file 6: Supplementary Table 6.** The QCI for ovarian cancer from 1990 to 2019 among different age groups.

## Data Availability

The primary data of cervical and ovarian cancer incidence, prevalence, mortality, DALYs, YLLs, and YLDs used in this study’s secondary analysis is extracted from the Global Burden of Disease Results Tool available in a public, open-access repository accessible from http://ghdx.healthdata.org/gbd-results-tool.
