# Supplementary material for: Global, regional, and national quality of care index of cervical and ovarian cancer: a systematic analysis for the global burden of disease study 1990–2019
Source: BMC Womens Health. 2024 Jan 25;24:69. doi: 10.1186/s12905-024-02884-9 (PMC10809627; doi:10.1186/s12905-024-02884-9)
Supplement: Supplementary file 1 — Additional file 1: Supplementary Table 1. All ages and age-standardized burden of cervical cancer from 1990 to 2019 in different locations. [file 12905_2024_2884_MOESM1_ESM.pdf]

Location type	Location name	Measure	Age, Metric	Burden				% Change (1990 to 2019)
				1990	2000	2010	2019	
Global		Incidence	All ages (number)	335642 (300354 to 393893)	399258 (360304 to 464773)	476523 (422622 to 520508)	565541 (481524 to 636435)	68.5 (43.8 to 89.9)
			Age-standardized rate (per 100,000)	14.9 (13.4 to 17.5)	14.1 (12.8 to 16.5)	13.5 (12 to 14.8)	13.4 (11.4 to 15)	-10.4 (-23.5 to 0.9)
		Prevalence	All ages (number)	1538196 (1390311 to 1779520)	1925623 (1739686 to 2181389)	2409890 (2113161 to 2563466)	2887521 (2439415 to 3219975)	87.7 (59.9 to 111.4)
			Age-standardized rate (per 100,000)	66.2 (60 to 76.5)	66.1 (59.8 to 74.9)	67.4 (59.1 to 71.7)	69.1 (58.3 to 77.1)	4.4 (-11.2 to 17.4)
		Deaths	All ages (number)	184527 (164836 to 218942)	208598 (189649 to 248707)	237933 (210986 to 268670)	280479 (238864 to 313930)	52 (29.5 to 74.1)
			Age-standardized rate (per 100,000)	8.5 (7.6 to 10.1)	7.7 (7 to 9.1)	6.8 (6.1 to 7.7)	6.5 (5.5 to 7.3)	-23.2 (-34.7 to -12.3)
		DALYs	All ages (number)	6176248 (5437672 to 7316926)	7000129 (6298899 to 8270869)	7817338 (6854644 to 8681430)	8955013 (7547733 to 9978462)	45 (22.9 to 67.7)
			Age-standardized rate (per 100,000)	275.1 (242.8 to 326.2)	248.9 (224.3 to 294.5)	221.4 (194.6 to 246.1)	210.6 (177.7 to 234.9)	-23.4 (-35.2 to -11.6)
		YLLs	All ages (number)	6041478 (5317181 to 7132260)	6835156 (6155204 to 8092317)	7614425 (6708007 to 8482394)	8712962 (7365279 to 9728886)	44.2 (22.2 to 67)
			Age-standardized rate (per 100,000)	269.2 (237.4 to 318.1)	243.2 (219.3 to 288.2)	215.6 (190.2 to 240.5)	204.9 (173.1 to 228.9)	-23.9 (-35.6 to -12.1)
		YLDs	All ages (number)	134770 (95241 to 180339)	164973 (116116 to 220267)	202913 (141128 to 268802)	242051 (171644 to 326024)	79.6 (52.1 to 102.2)
			Age-standardized rate (per 100,000)	5.9 (4.2 to 7.9)	5.8 (4.1 to 7.7)	5.7 (4 to 7.6)	5.7 (4.1 to 7.7)	-2.6 (-17.7 to 9.4)
World Bank Income Levels	World Bank High Income	Incidence	All ages (number)	74347 (68169 to 76654)	74654 (68471 to 76960)	76615 (69828 to 79185)	79094 (68439 to 88835)	6.4 (-3.9 to 18.2)
			Age-standardized rate (per 100,000)	12.1 (11 to 12.4)	10.7 (9.7 to 11)	9.7 (8.8 to 10)	9.2 (8 to 10.4)	-23.8 (-31.4 to -15.1)
		Prevalence	All ages (number)	416192 (375333 to 429603)	430712 (395885 to 443789)	452663 (407639 to 466786)	461242 (397233 to 519178)	10.8 (-0.6 to 23.9)
			Age-standardized rate (per 100,000)	71.7 (64.3 to 74)	65.8 (60.6 to 67.8)	62.5 (56.6 to 64.5)	59.9 (51.6 to 67.7)	-16.4 (-25.2 to -6.6)
		Deaths	All ages (number)	32524 (30220 to 33574)	31426 (28880 to 32489)	31285 (27682 to 32697)	33190 (29017 to 35666)	2 (-5.6 to 8.6)
			Age-standardized rate (per 100,000)	4.8 (4.5 to 5)	3.9 (3.6 to 4.1)	3.3 (3 to 3.4)	3.1 (2.7 to 3.3)	-36.5 (-40.1 to -32.5)
		DALYs	All ages (number)	941428 (868327 to 971198)	876378 (811450 to 903763)	836862 (768228 to 867512)	846454 (750866 to 906426)	-10.1 (-15 to -4.6)
			Age-standardized rate (per 100,000)	152.3 (139.4 to 157)	123.2 (113.4 to 127.2)	102.7 (95 to 106.4)	94.4 (85.2 to 100.8)	-38 (-41.1 to -34.4)

Location type	Location name	Measure	Age, Metric	Burden				% Change (1990 to 2019)
				1990	2000	2010	2019	
		YLLs	All ages (number)	907655 (837011 to 931492)	841647 (779461 to 865189)	800468 (729263 to 826915)	808967 (722369 to 866196)	-10.9 (-15.6 to -5.4)
			Age-standardized rate (per 100,000)	146.6 (134.2 to 150.3)	118.1 (108.7 to 121.1)	97.9 (89.8 to 101)	89.8 (81.7 to 95.8)	-38.8 (-41.7 to -35.2)
		YLDs	All ages (number)	33773 (24076 to 44127)	34731 (24861 to 45584)	36394 (25815 to 48334)	37487 (26094 to 50860)	11 (-0.2 to 24.1)
			Age-standardized rate (per 100,000)	5.6 (4 to 7.4)	5.1 (3.7 to 6.8)	4.8 (3.4 to 6.4)	4.6 (3.2 to 6.2)	-18.9 (-27.3 to -9.1)
	**World Bank Upper Middle Income**	Incidence	All ages (number)	119774 (108872 to 154954)	155390 (145030 to 185896)	199345 (165631 to 215199)	231109 (173791 to 267544)	93 (30.5 to 131.5)
			Age-standardized rate (per 100,000)	13.6 (12.3 to 17.5)	13.5 (12.6 to 16.2)	13.8 (11.5 to 14.8)	13.5 (10.1 to 15.6)	-0.5 (-32.5 to 19.1)
		Prevalence	All ages (number)	534948 (490294 to 674789)	768611 (716600 to 906661)	1053013 (862301 to 1138247)	1231817 (910963 to 1432202)	130.3 (54.1 to 175.9)
			Age-standardized rate (per 100,000)	57.3 (52.5 to 72.1)	63.3 (59.1 to 74.9)	71.3 (58.4 to 77)	73.9 (54.6 to 85.8)	29 (-13.1 to 54.6)
		Deaths	All ages (number)	68371 (61814 to 86597)	80129 (74956 to 97082)	95395 (79596 to 103092)	109690 (83717 to 126842)	60.4 (9.8 to 93.5)
			Age-standardized rate (per 100,000)	8.2 (7.4 to 10.4)	7.4 (6.9 to 9)	6.8 (5.7 to 7.3)	6.2 (4.7 to 7.1)	-24.6 (-47.9 to -9.2)
		DALYs	All ages (number)	2235214 (2018115 to 2855830)	2660082 (2489876 to 3199828)	3073608 (2536053 to 3321137)	3371381 (2536703 to 3905565)	50.8 (0.1 to 82.4)
			Age-standardized rate (per 100,000)	253.1 (228.7 to 323.6)	231.8 (217 to 279.3)	211.2 (174.7 to 227.9)	193.1 (145.5 to 224.2)	-23.7 (-49.2 to -7.9)
		YLLs	All ages (number)	2187863 (1971668 to 2802509)	2595252 (2431039 to 3130073)	2986867 (2459409 to 3233094)	3270174 (2462855 to 3797624)	49.5 (-1 to 81.4)
			Age-standardized rate (per 100,000)	247.9 (223.2 to 317.3)	226.3 (212.1 to 273.3)	205.3 (169.6 to 222)	187.1 (141.1 to 217.2)	-24.5 (-49.8 to -8.5)
		YLDs	All ages (number)	47351 (33596 to 65458)	64830 (45914 to 86910)	86742 (59652 to 116430)	101207 (67261 to 139387)	113.7 (42.2 to 155.1)
			Age-standardized rate (per 100,000)	5.2 (3.7 to 7.2)	5.5 (3.9 to 7.3)	5.9 (4.1 to 7.9)	6 (4 to 8.2)	14.4 (-24.2 to 36.7)
	**World Bank Lower Middle Income**	Incidence	All ages (number)	107184 (87171 to 126970)	128409 (107234 to 154786)	150822 (134798 to 183128)	190582 (163555 to 230583)	77.8 (53.5 to 116.8)
			Age-standardized rate (per 100,000)	16.9 (13.8 to 20)	15.6 (13.2 to 19)	13.8 (12.3 to 16.8)	13.6 (11.7 to 16.4)	-19.7 (-30.4 to -2.7)
		Prevalence	All ages (number)	454126 (366931 to 528510)	568131 (473085 to 674187)	701531 (616428 to 828344)	914779 (782925 to 1103853)	101.4 (72.7 to 146)
			Age-standardized rate (per 100,000)	65.1 (52.9 to 76.2)	62.8 (52.7 to 74.7)	59.1 (52.2 to 69.8)	61.5 (52.7 to 74.1)	-5.6 (-18.9 to 13.9)

Location type	Location name	Measure	Age, Metric	Burden				% Change (1990 to 2019)
				1990	2000	2010	2019	
		Deaths	All ages (number)	62397 (51084 to 75522)	72176 (61591 to 89288)	81528 (72266 to 100861)	100125 (84549 to 127255)	60.5 (37.4 to 96.7)
			Age-standardized rate (per 100,000)	10.7 (8.8 to 13)	9.7 (8.3 to 12.1)	8.1 (7.2 to 10.2)	7.6 (6.5 to 9.8)	-28.7 (-38.8 to -12.8)
		DALYs	All ages (number)	2223621 (1800188 to 2652481)	2562135 (2155707 to 3137253)	2845934 (2512572 to 3477982)	3404838 (2879036 to 4266168)	53.1 (31.1 to 89.9)
			Age-standardized rate (per 100,000)	346.4 (282.5 to 416.2)	310.1 (264 to 382.9)	259 (228.1 to 317.6)	241.6 (204.9 to 303.5)	-30.3 (-40.2 to -14.6)
		YLLs	All ages (number)	2182515 (1766789 to 2608779)	2511712 (2115675 to 3074021)	2784854 (2450837 to 3422542)	3326372 (2786056 to 4162231)	52.4 (30.2 to 89)
			Age-standardized rate (per 100,000)	340.2 (277.3 to 409.1)	304.2 (257.7 to 375.6)	253.6 (223.4 to 312.8)	236.1 (198.9 to 297.4)	-30.6 (-40.6 to -14.6)
		YLDs	All ages (number)	41106 (27802 to 55792)	50422 (34436 to 68733)	61080 (42592 to 83056)	78466 (54975 to 108816)	90.9 (63.7 to 133.2)
			Age-standardized rate (per 100,000)	6.2 (4.2 to 8.3)	5.9 (4 to 7.9)	5.4 (3.8 to 7.3)	5.4 (3.8 to 7.5)	-12.5 (-24.6 to 6.7)
	World Bank Low Income	Incidence	All ages (number)	34081 (26191 to 41629)	40483 (32203 to 48028)	49363 (37930 to 59779)	64322 (47789 to 80697)	88.7 (54 to 141.7)
			Age-standardized rate (per 100,000)	35.1 (27.2 to 42.8)	33 (26.3 to 39.1)	30.9 (23.7 to 37.4)	30.3 (22.8 to 37.8)	-13.7 (-29.1 to 8.7)
		Prevalence	All ages (number)	131736 (100136 to 161238)	156563 (123208 to 187252)	200809 (152370 to 242884)	277502 (208133 to 350744)	110.6 (68.5 to 178.7)
			Age-standardized rate (per 100,000)	121.2 (93.1 to 148)	112.7 (89.4 to 134)	110.3 (84.3 to 133.3)	114.9 (86.6 to 144.5)	-5.2 (-23.4 to 22.9)
		Deaths	All ages (number)	21098 (16294 to 25816)	24706 (19501 to 29240)	29535 (22380 to 35515)	37256 (28500 to 46704)	76.6 (45.8 to 122.8)
			Age-standardized rate (per 100,000)	23.8 (18.5 to 28.8)	22.2 (17.7 to 26.4)	20.6 (15.7 to 24.9)	19.6 (15.2 to 24.3)	-17.6 (-31.3 to 3)
		DALYs	All ages (number)	771244 (586155 to 943928)	895928 (705169 to 1073148)	1054496 (800138 to 1263621)	1325105 (996969 to 1670798)	71.8 (40.8 to 120.3)
			Age-standardized rate (per 100,000)	781.7 (604.1 to 960.8)	721.1 (569.8 to 856.9)	654.6 (495.4 to 783.7)	619.8 (471.6 to 778.9)	-20.7 (-34.8 to 0.5)
		YLLs	All ages (number)	758806 (576632 to 926402)	881073 (693044 to 1059078)	1035956 (784424 to 1245744)	1300397 (979930 to 1641838)	71.4 (40.4 to 119.7)
			Age-standardized rate (per 100,000)	769.5 (595.3 to 946.2)	709.6 (560 to 845.9)	643.7 (486.5 to 772.8)	608.8 (463.3 to 764.6)	-20.9 (-34.9 to 0.2)
		YLDs	All ages (number)	12438 (8169 to 17312)	14856 (9878 to 20244)	18540 (12383 to 26011)	24708 (15775 to 35941)	98.7 (60.4 to 159.3)
			Age-standardized rate (per 100,000)	12.2 (8.1 to 16.8)	11.5 (7.8 to 15.5)	11 (7.4 to 15.2)	11 (7.2 to 15.8)	-10 (-26.7 to 15.5)

Location type	Location name	Measure	Age, Metric	Burden				% Change (1990 to 2019)
				1990	2000	2010	2019	
SDI	High SDI	Incidence	All ages (number)	59690 (54298 to 61654)	59438 (55408 to 61571)	60840 (56418 to 63248)	63864 (55710 to 71455)	7 (-3.8 to 19)
			Age-standardized rate (per 100,000)	11.8 (10.7 to 12.2)	10.3 (9.6 to 10.6)	9.4 (8.7 to 9.8)	8.9 (7.7 to 10)	-24.7 (-32.4 to -16)
		Prevalence	All ages (number)	341394 (305243 to 353523)	349506 (325497 to 361078)	365112 (338917 to 380011)	377169 (326335 to 423849)	10.5 (-0.7 to 23.4)
			Age-standardized rate (per 100,000)	71.4 (63.9 to 73.9)	64.5 (59.6 to 66.4)	61 (56.1 to 63.5)	58.4 (50.7 to 65.6)	-18.2 (-26.5 to -8.5)
		Deaths	All ages (number)	25222 (23275 to 26193)	24200 (22248 to 25188)	24157 (21526 to 25464)	26173 (22823 to 28149)	3.8 (-4 to 10.1)
			Age-standardized rate (per 100,000)	4.6 (4.2 to 4.7)	3.7 (3.4 to 3.8)	3.1 (2.8 to 3.2)	2.9 (2.6 to 3.1)	-36.4 (-40 to -32.7)
		DALYs	All ages (number)	725847 (665204 to 752593)	671595 (632811 to 695958)	648913 (602428 to 678798)	672113 (608748 to 721998)	-7.4 (-12.1 to -2)
			Age-standardized rate (per 100,000)	143.2 (130.3 to 148.4)	114.4 (108.3 to 118.4)	96.4 (90 to 100.5)	89.7 (81.9 to 95.8)	-37.4 (-40.2 to -33.8)
		YLLs	All ages (number)	698355 (639923 to 720655)	643582 (605898 to 667883)	619711 (573282 to 649674)	641596 (580762 to 684914)	-8.1 (-12.9 to -2.8)
			Age-standardized rate (per 100,000)	137.6 (125.2 to 141.7)	109.4 (103.3 to 113.4)	91.8 (85.7 to 96)	85.3 (78.5 to 90.6)	-38 (-40.9 to -34.7)
		YLDs	All ages (number)	27493 (19697 to 36060)	28012 (19980 to 36885)	29202 (20764 to 38854)	30517 (21168 to 41571)	11 (-0.3 to 23.4)
			Age-standardized rate (per 100,000)	5.6 (4 to 7.3)	5 (3.5 to 6.6)	4.7 (3.3 to 6.2)	4.5 (3.1 to 6.1)	-20.4 (-28.7 to -10.9)
	High-middle SDI	Incidence	All ages (number)	75805 (71534 to 88876)	87464 (83470 to 102082)	103226 (89195 to 109523)	113123 (89780 to 129153)	49.2 (13.6 to 72)
			Age-standardized rate (per 100,000)	12.8 (12 to 15)	12.2 (11.7 to 14.3)	12.1 (10.4 to 12.8)	11.6 (9.2 to 13.2)	-9.3 (-31.3 to 4.7)
		Prevalence	All ages (number)	353662 (334470 to 411171)	439388 (419062 to 510074)	560745 (479013 to 593809)	618791 (483509 to 710638)	75 (31.5 to 103.8)
			Age-standardized rate (per 100,000)	60 (56.8 to 69.9)	61.8 (58.9 to 71.6)	67 (57.2 to 71)	66.8 (51.9 to 76.8)	11.3 (-16.6 to 29.6)
		Deaths	All ages (number)	41353 (38694 to 48405)	44219 (41877 to 51981)	47527 (42204 to 50531)	51771 (41664 to 57874)	25.2 (-3.5 to 43.3)
			Age-standardized rate (per 100,000)	6.9 (6.5 to 8.1)	6.2 (5.8 to 7.2)	5.4 (4.8 to 5.7)	4.9 (3.9 to 5.5)	-29.7 (-46.2 to -19.5)
		DALYs	All ages (number)	1274572 (1193740 to 1497010)	1387196 (1322053 to 1650982)	1474177 (1292594 to 1562334)	1543704 (1235997 to 1729870)	21.1 (-9.5 to 39.8)
			Age-standardized rate (per 100,000)	215.2 (201.5 to 252.9)	194.2 (185 to 231.3)	171 (149.9 to 181)	154.7 (124 to 173.5)	-28.1 (-46.5 to -17)

Location type	Location name	Measure	Age, Metric	Burden				% Change (1990 to 2019)
				1990	2000	2010	2019	
		YLLs	All ages (number)	1243688 (1162927 to 1465147)	1350124 (1285002 to 1605131)	1428217 (1254093 to 1515498)	1492922 (1191878 to 1676854)	20 (-10.5 to 38.7)
			Age-standardized rate (per 100,000)	210 (196.1 to 247.5)	189 (179.8 to 224.8)	165.6 (145.4 to 175.6)	149.4 (119.1 to 167.6)	-28.9 (-47.1 to -17.8)
		YLDs	All ages (number)	30883 (21993 to 41513)	37072 (26650 to 49094)	45960 (32225 to 61296)	50782 (34921 to 69817)	64.4 (24.2 to 90.6)
			Age-standardized rate (per 100,000)	5.2 (3.7 to 7)	5.2 (3.7 to 6.9)	5.4 (3.8 to 7.3)	5.3 (3.7 to 7.3)	2 (-23.4 to 18.5)
	Middle SDI	Incidence	All ages (number)	92178 (81453 to 116400)	121451 (108533 to 142557)	154385 (127874 to 166331)	183337 (144492 to 208859)	98.9 (53 to 134.4)
			Age-standardized rate (per 100,000)	14.9 (13.2 to 18.9)	14.5 (13 to 17)	14 (11.7 to 15.1)	13.4 (10.6 to 15.3)	-9.6 (-30.3 to 6.3)
		Prevalence	All ages (number)	409753 (362681 to 502752)	588650 (523035 to 683745)	785320 (645117 to 850090)	946469 (735439 to 1079372)	131 (77.4 to 172.1)
			Age-standardized rate (per 100,000)	60.1 (53.2 to 74)	64.3 (57.2 to 74.6)	67.2 (55.3 to 72.6)	68.4 (53.2 to 78.1)	13.8 (-12.6 to 34)
		Deaths	All ages (number)	52526 (46634 to 65121)	63665 (57459 to 74729)	76964 (63888 to 84001)	90100 (71333 to 103200)	71.5 (30.9 to 105.8)
			Age-standardized rate (per 100,000)	9.3 (8.3 to 11.5)	8.4 (7.6 to 9.9)	7.6 (6.3 to 8.3)	6.8 (5.4 to 7.8)	-27.2 (-44.1 to -13.3)
		DALYs	All ages (number)	1790629 (1588481 to 2223171)	2164020 (1951420 to 2528304)	2517393 (2074153 to 2740601)	2817246 (2223191 to 3217721)	57.3 (19.4 to 88.8)
			Age-standardized rate (per 100,000)	287.8 (255 to 356.3)	259.4 (234.7 to 303.1)	228.5 (188.3 to 249)	204.6 (161.9 to 233.5)	-28.9 (-45.9 to -14.6)
		YLLs	All ages (number)	1754415 (1556461 to 2176056)	2113978 (1903473 to 2479655)	2451660 (2020227 to 2668279)	2738503 (2157802 to 3126816)	56.1 (18.2 to 87.1)
			Age-standardized rate (per 100,000)	282.2 (251 to 350.2)	253.6 (228.5 to 297.1)	222.7 (183.9 to 242.8)	198.9 (157.4 to 226.8)	-29.5 (-46.4 to -15.5)
		YLDs	All ages (number)	36214 (25547 to 48839)	50041 (34809 to 67284)	65733 (44992 to 87804)	78743 (55202 to 106370)	117.4 (66.1 to 155.7)
			Age-standardized rate (per 100,000)	5.6 (4 to 7.5)	5.7 (4 to 7.7)	5.8 (4 to 7.7)	5.7 (4 to 7.7)	2.4 (-22.1 to 20.4)
	Low-middle SDI	Incidence	All ages (number)	66216 (54058 to 81763)	81246 (69141 to 98740)	97367 (86682 to 116126)	125963 (107883 to 150105)	90.2 (64.4 to 137)
			Age-standardized rate (per 100,000)	18 (14.9 to 22.5)	17.1 (14.7 to 21)	15.5 (13.9 to 18.7)	15.8 (13.6 to 18.9)	-12.5 (-24.3 to 8.4)
		Prevalence	All ages (number)	274902 (223505 to 330393)	355700 (300422 to 420226)	449014 (396402 to 521536)	601452 (512137 to 711932)	118.8 (87.8 to 174.9)
			Age-standardized rate (per 100,000)	67.5 (55 to 82)	67.6 (57.3 to 80.6)	66.1 (58.7 to 77.3)	71.7 (61 to 84.8)	6.3 (-8.6 to 32.8)

Location type	Location name	Measure	Age, Metric	Burden 1990	Burden 2000	Burden 2010	Burden 2019	% Change (1990 to 2019)
		**Deaths**	All ages (number)	39209 (32461 to 50054)	45839 (39229 to 57786)	53065 (47121 to 65218)	66678 (57270 to 81245)	70.1 (46.3 to 108.4)
			Age-standardized rate (per 100,000)	11.7 (9.7 to 15.1)	10.6 (9.1 to 13.5)	9.2 (8.1 to 11.4)	8.9 (7.6 to 10.8)	-24.4 (-35 to -7.9)
		**DALYs**	All ages (number)	1419286 (1160748 to 1789128)	1650582 (1391854 to 2033803)	1865163 (1650493 to 2243632)	2282245 (1948327 to 2722926)	60.8 (37.7 to 98.2)
			Age-standardized rate (per 100,000)	381.9 (315.3 to 485.5)	345.2 (293.6 to 429.7)	296.5 (261.9 to 359.4)	285.6 (244.6 to 342.2)	-25.2 (-35.9 to -8.2)
		**YLLs**	All ages (number)	1394225 (1141664 to 1752554)	1618960 (1360482 to 1999453)	1826073 (1616822 to 2204624)	2230841 (1902939 to 2671047)	60 (36.5 to 96.8)
			Age-standardized rate (per 100,000)	375.4 (309.4 to 475.9)	338.9 (289.4 to 423.5)	290.5 (258 to 353.4)	279.4 (239 to 335.8)	-25.6 (-36.5 to -8.6)
		**YLDs**	All ages (number)	25061 (16946 to 33783)	31621 (21762 to 43070)	39090 (27351 to 52678)	51404 (36161 to 69166)	105.1 (75 to 154)
			Age-standardized rate (per 100,000)	6.5 (4.4 to 8.8)	6.3 (4.4 to 8.5)	6 (4.2 to 8.1)	6.3 (4.4 to 8.4)	-3.4 (-17.5 to 19.5)
	**Low SDI**	**Incidence**	All ages (number)	41499 (31775 to 50802)	49339 (39352 to 58765)	60329 (47587 to 71953)	78821 (61613 to 97925)	89.9 (57.7 to 145.1)
			Age-standardized rate (per 100,000)	27.7 (21.6 to 34.3)	26 (20.8 to 31)	23.9 (18.9 to 28.6)	23.2 (18.3 to 28.8)	-16.3 (-29.7 to 7.1)
		**Prevalence**	All ages (number)	157299 (119099 to 190701)	190779 (151323 to 227320)	247832 (193749 to 293642)	341465 (265064 to 428747)	117.1 (78.7 to 188.5)
			Age-standardized rate (per 100,000)	92.7 (70.8 to 112.5)	87.5 (70 to 104)	84.9 (66.8 to 100.8)	88 (68.9 to 110.1)	-5 (-21.5 to 23.3)
		**Deaths**	All ages (number)	26080 (20234 to 32111)	30513 (24277 to 36886)	36029 (28563 to 42897)	45540 (35797 to 56258)	74.6 (46.8 to 121.4)
			Age-standardized rate (per 100,000)	19.2 (15 to 23.7)	17.9 (14.3 to 21.5)	16 (12.8 to 19.2)	15.1 (11.9 to 18.5)	-21.5 (-33.8 to -1.6)
		**DALYs**	All ages (number)	961196 (732588 to 1179639)	1121149 (886046 to 1358141)	1305272 (1037504 to 1549467)	1632490 (1271609 to 2044290)	69.8 (42 to 119.9)
			Age-standardized rate (per 100,000)	630.6 (487.6 to 777.4)	581.7 (463.2 to 702.7)	510.5 (406.8 to 607.9)	477.5 (374.3 to 591.4)	-24.3 (-36.4 to -3.8)
		**YLLs**	All ages (number)	946178 (718554 to 1161698)	1103056 (872549 to 1340887)	1282501 (1019415 to 1520776)	1602067 (1248560 to 2008867)	69.3 (41.4 to 119.2)
			Age-standardized rate (per 100,000)	621.1 (480.3 to 763.3)	572.7 (455.9 to 694.3)	502.1 (399.8 to 596.7)	469.1 (367.5 to 580.4)	-24.5 (-36.7 to -4)
		**YLDs**	All ages (number)	15018 (9896 to 20933)	18093 (12071 to 24888)	22771 (15391 to 31400)	30423 (20152 to 43752)	102.6 (66.9 to 167.3)
			Age-standardized rate (per 100,000)	9.5 (6.3 to 13.2)	9 (6 to 12.3)	8.4 (5.8 to 11.6)	8.4 (5.7 to 12)	-11.2 (-26.2 to 15)

Location type	Location name	Measure	Age, Metric	Burden				% Change (1990 to 2019)
				1990	2000	2010	2019	
WHO Regions	African Region	Incidence	All ages (number)	46065 (36560 to 55883)	58289 (47557 to 68995)	72664 (57966 to 85222)	93772 (72680 to 114333)	103.6 (65.6 to 151.8)
			Age-standardized rate (per 100,000)	32.4 (25.7 to 39.3)	31.6 (25.9 to 37.1)	29.5 (23.7 to 34.4)	27.9 (21.9 to 33.9)	-14.1 (-29.5 to 5.1)
		Prevalence	All ages (number)	182348 (144498 to 220461)	230149 (186375 to 273094)	301916 (240468 to 355030)	414235 (319056 to 512991)	127.2 (82.5 to 187.7)
			Age-standardized rate (per 100,000)	113.7 (90.1 to 137.9)	108 (87.9 to 127.7)	104.9 (84 to 123.1)	106.5 (82.7 to 131)	-6.3 (-24.3 to 17)
		Deaths	All ages (number)	28179 (22531 to 34636)	35168 (28772 to 41812)	42902 (34384 to 50482)	53396 (41571 to 64892)	89.5 (56.2 to 131.2)
			Age-standardized rate (per 100,000)	21.8 (17.4 to 27)	21.3 (17.4 to 25)	19.7 (16 to 23.1)	18.1 (14.3 to 21.8)	-17.2 (-31.2 to 0.3)
		DALYs	All ages (number)	1018788 (806970 to 1250739)	1262197 (1022438 to 1511487)	1519887 (1219597 to 1807544)	1878932 (1455936 to 2310125)	84.4 (50.1 to 129.4)
			Age-standardized rate (per 100,000)	710.7 (566.7 to 873.5)	677.3 (552.9 to 806.7)	610.6 (488.8 to 720.7)	555 (430.3 to 676.8)	-21.9 (-35.8 to -4.5)
		YLLs	All ages (number)	1001801 (795387 to 1231598)	1240598 (1005722 to 1483559)	1492283 (1195696 to 1774850)	1842470 (1427759 to 2266402)	83.9 (49.7 to 128.9)
			Age-standardized rate (per 100,000)	699.4 (559.2 to 857.6)	666.3 (542.4 to 792.7)	600.1 (480.1 to 709.1)	544.8 (422.6 to 666.1)	-22.1 (-35.9 to -4.7)
		YLDs	All ages (number)	16986 (11170 to 23760)	21599 (14549 to 29364)	27604 (18543 to 37569)	36462 (24021 to 51877)	114.7 (72.8 to 167.9)
			Age-standardized rate (per 100,000)	11.3 (7.5 to 15.8)	11 (7.4 to 14.8)	10.4 (7.1 to 14.1)	10.1 (6.8 to 14.3)	-10.7 (-27.2 to 10.2)
	Eastern Mediterranean Region	Incidence	All ages (number)	7690 (5770 to 9010)	10627 (8445 to 12382)	13990 (11146 to 16659)	18394 (14422 to 22642)	139.2 (99.1 to 219.9)
			Age-standardized rate (per 100,000)	7.2 (5.4 to 8.5)	7.5 (5.9 to 8.7)	7.1 (5.7 to 8.4)	6.9 (5.5 to 8.4)	-4.2 (-19.5 to 27.4)
		Prevalence	All ages (number)	32045 (24354 to 37747)	46340 (36402 to 53956)	65026 (51265 to 77543)	90333 (69597 to 112228)	181.9 (135.6 to 277)
			Age-standardized rate (per 100,000)	26.8 (20.2 to 31.4)	28.7 (22.7 to 33.2)	28.9 (22.6 to 34.3)	30.1 (23.4 to 36.9)	12.5 (-5.6 to 51.2)
		Deaths	All ages (number)	4582 (3418 to 5412)	6103 (4850 to 7176)	7595 (6118 to 8915)	9444 (7456 to 11530)	106.1 (71.7 to 171.6)
			Age-standardized rate (per 100,000)	4.7 (3.5 to 5.7)	4.8 (3.8 to 5.6)	4.4 (3.6 to 5.2)	4.1 (3.2 to 4.9)	-14.6 (-28.3 to 13)
		DALYs	All ages (number)	160474 (119873 to 189483)	216761 (170489 to 256279)	268401 (212675 to 315833)	331307 (254792 to 407536)	106.5 (70.7 to 172.9)
			Age-standardized rate (per 100,000)	149.1 (111.3 to 176.3)	151.4 (120.1 to 178.7)	136.2 (109.3 to 160.1)	124.2 (97.8 to 152)	-16.7 (-30.6 to 9.6)

Location type	Location name	Measure	Age, Metric	Burden 1990	Burden 2000	Burden 2010	Burden 2019	% Change (1990 to 2019)
		YLLs	All ages (number)	157465 (117777 to 185853)	212516 (166784 to 251590)	262649 (207935 to 309662)	323532 (249106 to 399272)	105.5 (69.5 to 171.4)
			Age-standardized rate (per 100,000)	146.4 (109.7 to 173.7)	148.6 (117.9 to 175.1)	133.4 (106.9 to 157.1)	121.4 (95.7 to 148.6)	-17.1 (-30.9 to 9.5)
		YLDs	All ages (number)	3009 (1946 to 4089)	4244 (2800 to 5724)	5753 (3847 to 7957)	7775 (5193 to 10769)	158.4 (114.2 to 247.2)
			Age-standardized rate (per 100,000)	2.7 (1.8 to 3.6)	2.8 (1.9 to 3.8)	2.8 (1.9 to 3.8)	2.8 (1.9 to 3.8)	3 (-14.5 to 38.9)
	European Region	Incidence	All ages (number)	73343 (65986 to 76160)	73433 (69082 to 75411)	74887 (66709 to 77105)	73345 (63335 to 83205)	0 (-9.8 to 11.2)
			Age-standardized rate (per 100,000)	13.7 (12.3 to 14.2)	12.7 (11.9 to 13)	12 (10.6 to 12.4)	11.1 (9.5 to 12.6)	-18.9 (-27 to -9.5)
		Prevalence	All ages (number)	363681 (325488 to 378790)	379171 (355366 to 389898)	410199 (356860 to 421703)	406568 (344440 to 462326)	11.8 (0 to 25.3)
			Age-standardized rate (per 100,000)	73.1 (65.7 to 76.3)	70.5 (66 to 72.6)	71.4 (61.9 to 73.3)	67.8 (57.1 to 77.2)	-7.3 (-17 to 4.2)
		Deaths	All ages (number)	37517 (34124 to 38958)	35926 (33817 to 37011)	34124 (31213 to 35375)	33081 (29639 to 36531)	-11.8 (-18 to -4.3)
			Age-standardized rate (per 100,000)	6.4 (5.8 to 6.6)	5.6 (5.3 to 5.7)	4.8 (4.4 to 4.9)	4.2 (3.7 to 4.6)	-34.2 (-39.3 to -28.2)
		DALYs	All ages (number)	1083634 (979019 to 1128064)	1042460 (989986 to 1072900)	982089 (884668 to 1013123)	918838 (811157 to 1016627)	-15.2 (-22 to -7.3)
			Age-standardized rate (per 100,000)	200.6 (180.7 to 208.9)	178.4 (168.8 to 183.7)	154.7 (138.5 to 159.5)	135.2 (118.8 to 150)	-32.6 (-38.4 to -26)
		YLLs	All ages (number)	1052689 (949876 to 1092496)	1010649 (963932 to 1036172)	948429 (858773 to 975547)	885494 (787659 to 978687)	-15.9 (-22.7 to -8.1)
			Age-standardized rate (per 100,000)	194.6 (174.9 to 202)	172.7 (164.4 to 177)	149.1 (133.9 to 153.3)	130 (114.8 to 144.1)	-33.2 (-39.1 to -26.7)
		YLDs	All ages (number)	30945 (22035 to 40619)	31811 (22848 to 41621)	33660 (24215 to 44562)	33343 (23124 to 45816)	7.8 (-3.8 to 20.3)
			Age-standardized rate (per 100,000)	6 (4.3 to 7.9)	5.7 (4.1 to 7.5)	5.6 (4 to 7.5)	5.3 (3.6 to 7.3)	-12 (-22.1 to -1.2)
	Region of the Americas	Incidence	All ages (number)	63050 (59528 to 66271)	77136 (72176 to 80394)	84900 (80994 to 90604)	99344 (86452 to 113504)	57.6 (38 to 80)
			Age-standardized rate (per 100,000)	18.9 (17.8 to 19.8)	18.4 (17.1 to 19.1)	16.4 (15.7 to 17.5)	16.4 (14.2 to 18.7)	-13.2 (-24.2 to -0.6)
		Prevalence	All ages (number)	318552 (302691 to 333010)	408444 (381466 to 424271)	456600 (437745 to 484627)	538789 (465176 to 618654)	69.1 (46.5 to 94.7)
			Age-standardized rate (per 100,000)	94.2 (89.6 to 98.5)	96.7 (90.3 to 100.5)	89.8 (86.1 to 95.4)	92.7 (79.8 to 106.5)	-1.6 (-14.9 to 13.5)

Location type	Location name	Measure	Age, Metric	Burden				% Change (1990 to 2019)
				1990	2000	2010	2019	
		Deaths	All ages (number)	31450 (29506 to 33390)	36504 (33973 to 37947)	39585 (37212 to 42614)	45880 (41564 to 50779)	45.9 (33.4 to 60.5)
			Age-standardized rate (per 100,000)	9.5 (8.9 to 10.1)	8.7 (8.1 to 9)	7.4 (7 to 8)	7.1 (6.4 to 7.8)	-25.7 (-32.1 to -18.1)
		DALYs	All ages (number)	1045599 (990449 to 1109391)	1187213 (1106031 to 1233690)	1251029 (1188945 to 1344670)	1412411 (1274478 to 1573926)	35.1 (22.4 to 50)
			Age-standardized rate (per 100,000)	317.1 (300.6 to 336.1)	284.6 (264.7 to 295.6)	240.9 (228.9 to 259)	230.9 (208.1 to 257.4)	-27.2 (-34.1 to -19)
		YLLs	All ages (number)	1019138 (964988 to 1085518)	1153991 (1077381 to 1197784)	1213969 (1152506 to 1308492)	1368848 (1234552 to 1524455)	34.3 (21.6 to 49.1)
			Age-standardized rate (per 100,000)	309.2 (292.8 to 328.9)	276.7 (258 to 287.3)	233.7 (221.7 to 251.9)	223.6 (201.3 to 249.3)	-27.7 (-34.6 to -19.7)
		YLDs	All ages (number)	26462 (19048 to 34822)	33222 (23729 to 43540)	37060 (26824 to 48782)	43563 (30364 to 58147)	64.6 (43.5 to 89.2)
			Age-standardized rate (per 100,000)	7.9 (5.7 to 10.4)	7.9 (5.6 to 10.3)	7.2 (5.2 to 9.5)	7.3 (5.1 to 9.8)	-7.1 (-19.3 to 7.3)
	South-East Asia Region	Incidence	All ages (number)	77333 (60084 to 93848)	92658 (76666 to 114311)	102156 (89589 to 131182)	128159 (106048 to 159948)	65.7 (35.4 to 113.7)
			Age-standardized rate (per 100,000)	17.5 (13.9 to 21.6)	16 (13.4 to 20.1)	13.2 (11.6 to 17.1)	13.1 (10.8 to 16.2)	-25.6 (-39 to -4.9)
		Prevalence	All ages (number)	334042 (258222 to 400812)	422608 (343639 to 505867)	482935 (421016 to 604944)	618740 (507269 to 761796)	85.2 (51.1 to 141.1)
			Age-standardized rate (per 100,000)	67.9 (52.8 to 81.8)	66.1 (54.1 to 80)	58.2 (50.7 to 73.2)	60.6 (49.7 to 74.6)	-10.8 (-27.2 to 15.1)
		Deaths	All ages (number)	44208 (34934 to 54548)	50697 (42301 to 65476)	54297 (47005 to 73436)	66891 (54920 to 89489)	51.3 (22.7 to 95.6)
			Age-standardized rate (per 100,000)	11.1 (8.9 to 13.8)	9.7 (8.2 to 12.7)	7.6 (6.6 to 10.4)	7.2 (5.9 to 9.6)	-35.5 (-47.4 to -17.7)
		DALYs	All ages (number)	1623382 (1271686 to 1976875)	1831860 (1517776 to 2318832)	1903618 (1645638 to 2520720)	2258636 (1842723 to 2972147)	39.1 (12.5 to 80.6)
			Age-standardized rate (per 100,000)	363.4 (286.3 to 446.6)	315.1 (261.9 to 403.9)	245.3 (212.2 to 327.3)	229.2 (187.1 to 302.5)	-36.9 (-48.9 to -18.8)
		YLLs	All ages (number)	1593560 (1247643 to 1937821)	1795072 (1483664 to 2263295)	1861969 (1614083 to 2464429)	2205734 (1792983 to 2909564)	38.4 (11.6 to 80.4)
			Age-standardized rate (per 100,000)	357 (280.8 to 439.6)	309 (256.9 to 394.7)	240.1 (208.1 to 322.2)	223.9 (182.7 to 295.6)	-37.3 (-49.3 to -18.9)
		YLDs	All ages (number)	29823 (19565 to 40551)	36788 (25213 to 50098)	41649 (29206 to 57548)	52902 (36466 to 74683)	77.4 (44.7 to 129)
			Age-standardized rate (per 100,000)	6.4 (4.3 to 8.7)	6.1 (4.2 to 8.2)	5.2 (3.7 to 7.2)	5.3 (3.7 to 7.4)	-17.7 (-32.3 to 6.2)

Location type	Location name	Measure	Age, Metric	Burden 1990	Burden 2000	Burden 2010	Burden 2019	% Change (1990 to 2019)
	Western Pacific Region	Incidence	All ages (number)	67296 (56912 to 102906)	86100 (77576 to 111781)	126743 (94289 to 141743)	151207 (94922 to 184177)	124.7 (16.3 to 198.3)
			Age-standardized rate (per 100,000)	10.1 (8.6 to 15.4)	9.7 (8.8 to 12.7)	11.3 (8.4 to 12.6)	11.5 (7.2 to 14)	13.8 (-41 to 50.6)
		Prevalence	All ages (number)	303435 (259665 to 450269)	433638 (389358 to 551112)	686965 (510299 to 766235)	811996 (500678 to 991572)	167.6 (39.5 to 251.3)
			Age-standardized rate (per 100,000)	43.2 (37.1 to 64)	46.9 (42.3 to 59.5)	60.9 (45.4 to 68)	64.4 (39.9 to 78.7)	49 (-22.5 to 95)
		Deaths	All ages (number)	38127 (31712 to 56698)	43703 (38996 to 57696)	58861 (44002 to 65891)	71137 (45603 to 86835)	86.6 (-4.7 to 152.6)
			Age-standardized rate (per 100,000)	6 (5 to 8.9)	5.2 (4.6 to 6.9)	5.3 (4 to 5.9)	5.1 (3.3 to 6.2)	-15.8 (-56.6 to 13.8)
		DALYs	All ages (number)	1229848 (1008052 to 1849217)	1444054 (1287961 to 1911571)	1874815 (1355919 to 2106126)	2135723 (1333747 to 2632365)	73.7 (-15.5 to 135.8)
			Age-standardized rate (per 100,000)	184 (151.5 to 276.2)	163.6 (146.2 to 215.8)	166.5 (121 to 187.1)	158.2 (98.9 to 194.8)	-14 (-57.9 to 17.1)
		YLLs	All ages (number)	1202656 (985583 to 1806326)	1407184 (1254014 to 1868500)	1818152 (1312237 to 2047350)	2068297 (1280171 to 2561814)	72 (-16.7 to 134.9)
			Age-standardized rate (per 100,000)	180 (148 to 270.7)	159.5 (142.2 to 211)	161.5 (116.8 to 181.9)	152.9 (95.2 to 189.4)	-15 (-58.6 to 16.4)
		YLDs	All ages (number)	27192 (18440 to 40069)	36869 (25604 to 50762)	56663 (36471 to 77703)	67426 (40108 to 95176)	148 (28 to 226.4)
			Age-standardized rate (per 100,000)	4 (2.7 to 5.8)	4.1 (2.8 to 5.6)	5 (3.3 to 6.9)	5.2 (3.1 to 7.4)	30.9 (-32.6 to 72.2)

Data in parentheses are 95% Uncertainty Intervals (95% UIs); DALYs = Disability-Adjusted Life Years; YLLs = Years of Life Lost; YLDs = Years Lived with Disability
