# Supplementary material for: Global, regional, and national quality of care index of cervical and ovarian cancer: a systematic analysis for the global burden of disease study 1990–2019
Source: BMC Womens Health. 2024 Jan 25;24:69. doi: 10.1186/s12905-024-02884-9 (PMC10809627; doi:10.1186/s12905-024-02884-9)
Supplement: Supplementary file 2 — Additional file 2: Supplementary Table 2. The QCI for cervical cancer from 1990 to 2019 in different locations. [file 12905_2024_2884_MOESM2_ESM.pdf]

Location type	Location name	QCI				% Change (1990 to 2019)
		1990	2000	2010	2019	
Global		43.1	48.3	54.9	58.5	35.8
World Bank Income Levels	World Bank High Income	73.7	78.4	82.9	84.3	14.4
	World Bank Upper Middle Income	38.6	48.7	59	64.6	67.6
	World Bank Lower Middle Income	29.4	33.4	39.8	45.5	54.9
	World Bank Low Income	18.1	18.7	22.6	27.9	54.4
SDI	High SDI	75.8	80.2	84.3	85.2	12.4
	High-middle SDI	49.7	56.5	66.2	70.2	41.4
	Middle SDI	34.1	43.3	51.7	57.8	69.3
	Low-middle SDI	25.7	31.7	38.7	45.1	75.2
	Low SDI	14.7	16.1	21.8	27.6	87.8
WHO Regions	African Region	19.7	19.5	23.3	29.2	48.0
	Eastern Mediterranean Region	26.7	29.9	35.7	42.6	59.4
	European Region	62.7	66.7	73.4	76.6	22.1
	Region of the Americas	54.3	60.2	64.3	67.7	24.6
	South-East Asia Region	29	35.4	42.1	47.7	64.1
	Western Pacific Region	40.6	51.5	62.9	67.4	65.8
Country	Afghanistan	12.3	11.3	17.7	23.8	92.7
	Albania	60.3	66.6	71.5	75.4	24.9
	Algeria	48	53	56.9	63.6	32.6
	American Samoa	46.2	53.9	54.4	56	21.1
	Andorra	76.5	84.4	86.4	85.5	11.7
	Angola	9.6	11.1	19.7	26.9	182.0
	Antigua and Barbuda	54.3	52.9	57.5	58.8	8.2
	Argentina	61.9	65.9	68.8	73.8	19.2
	Armenia	55.4	58.1	62.5	65.1	17.5
	Australia	88.6	93.2	94.7	95.8	8.2
	Austria	73.5	78.9	80	80.8	9.9
	Azerbaijan	49	47.3	51.6	56.3	14.9
	Bahamas	53	60.5	62	63.2	19.3
	Bahrain	35.7	46.8	51.6	58.9	65.0
	Bangladesh	19.2	24.2	35.1	45.6	137.9
	Barbados	52.6	59.5	63.2	63.8	21.4
	Belarus	55.3	59.7	68.6	75.4	36.3
	Belgium	69.5	77.6	80.9	81.6	17.4
	Belize	39.2	48.1	54.7	57	45.2
	Benin	15.9	19.7	23	25.9	62.5
	Bermuda	57	66.4	74.4	77.3	35.6
	Bhutan	17.9	28.9	40	47.3	163.6
	Bolivia (Plurinational State of)	20.5	30.7	38.5	45.2	120.2
	Bosnia and Herzegovina	53.9	60.9	68.4	70.9	31.6
	Botswana	28.3	30.9	36.6	46.5	64.4
	Brazil	38.7	47.1	54.3	59.7	54.1
	Brunei Darussalam	64.2	61.9	67.7	71.5	11.4
	Bulgaria	70.9	72.2	74	77.7	9.5
	Burkina Faso	15.9	20	24.6	26.5	66.8
	Burundi	11.8	12.3	20.9	22.6	91.6
	Cabo Verde	35	36.2	44.1	45	28.5
	Cambodia	24.9	31.8	40.8	48.9	96.0

Location type	Location name	QCI				% Change (1990 to 2019)
		1990	2000	2010	2019	
	Cameroon	19	19.2	22.9	30.9	62.4
	Canada	92.3	95.8	98.1	99.4	7.7
	Central African Republic	7.1	3.2	6.9	7.7	7.3
	Chad	12.1	9.7	12.1	15.9	31.4
	Chile	55.6	62.5	70.2	75.2	35.2
	China	21.6	41.3	58	64.1	196.9
	Colombia	37.2	48.5	59.5	65.8	76.6
	Comoros	13.3	17.3	21.7	29.6	121.5
	Congo	15.3	13.6	24	28.9	89.2
	Cook Islands	61.1	65.1	67.6	69.8	14.4
	Costa Rica	52.3	55.3	64.6	68.9	31.7
	Croatia	80.9	85.2	87.2	89.3	10.4
	Cuba	62.6	71.5	71.7	74	18.3
	Cyprus	53.8	66.1	74.1	72.4	34.5
	Czechia	63.5	70.9	73.8	76.1	19.9
	Côte d'Ivoire	19	17.8	22.5	25.2	32.7
	Democratic People's Republic of Korea	59.6	56.2	60.3	63.9	7.2
	Democratic Republic of the Congo	13.7	12.6	14.7	21.7	59.0
	Denmark	66.8	72.1	77.8	80.3	20.2
	Djibouti	17.8	14.9	22.5	30.6	71.8
	Dominica	49.7	57.8	55.8	54.3	9.3
	Dominican Republic	35.6	48.9	51.4	54.2	52.2
	Ecuador	35.8	50.1	55.6	61.1	70.8
	Egypt	27.2	31.5	34.2	42.1	54.9
	El Salvador	31.2	44.7	51.9	54.5	74.4
	Equatorial Guinea	5.2	15.7	31.4	36.9	612.8
	Eritrea	3.6	7.7	12.7	20.5	463.3
	Estonia	70.3	76.9	84	87.5	24.4
	Eswatini	22.6	17	17.4	28.8	27.4
	Ethiopia	7.8	8.9	15.7	23.5	201.4
	Fiji	42	43.8	45	48.9	16.6
	Finland	71.4	82	84	84.3	18.1
	France	70.4	79.1	83.3	84	19.3
	Gabon	23.4	23.7	29.4	36.1	54.5
	Gambia	21.5	22.9	25	29.5	37.4
	Georgia	63.9	66.9	63	63.6	-0.4
	Germany	79.2	80.2	85.7	88.1	11.2
	Ghana	26.8	29.9	32.1	36.8	37.2
	Greece	73	76.7	81.3	81.6	11.7
	Greenland	58.7	63.6	72.5	76.8	30.9
	Grenada	45.9	55.4	55.9	57.8	25.9
	Guam	54.4	68.5	69.5	68.1	25.3
	Guatemala	18.7	32.6	38.8	41.5	121.6
	Guinea	11.9	13.5	17.9	21.3	79.2
	Guinea-Bissau	14.9	15.2	19.5	23.1	54.9
	Guyana	33	43.3	42.6	47.8	45.1
	Haiti	14.1	18.6	21.2	29.3	108.4
	Honduras	32.8	36.6	40.3	41.7	27.1

Location type	Location name	QCI				% Change (1990 to 2019)
		1990	2000	2010	2019	
	Hungary	70.7	76	75.9	78.6	11.3
	Iceland	77.6	82.8	84.1	85.2	9.8
	India	24.2	29.4	37.7	44.2	82.5
	Indonesia	34.4	40.4	44.4	48.4	40.7
	Iran (Islamic Republic of)	43.6	52.9	53.4	59.4	36.4
	Iraq	36.9	39.8	48.9	56.2	52.5
	Ireland	76.2	82.1	87.9	89.4	17.3
	Israel	64.6	71.7	76.6	78.5	21.6
	Italy	77.2	84.2	89.3	90.4	17.1
	Jamaica	48.8	56.3	61.6	62	27.2
	Japan	82.4	86.4	94.1	95.4	15.8
	Jordan	41	47.5	55.7	60.4	47.4
	Kazakhstan	49.8	49.4	58.9	69.4	39.5
	Kenya	20.6	25	16.6	24.5	18.6
	Kiribati	24.3	28.3	29.3	31	27.4
	Kuwait	58.8	64.2	60.3	65.4	11.1
	Kyrgyzstan	47.2	46.3	54	57.9	22.6
	Lao People's Democratic Republic	19.2	25.9	35	42.6	122.1
	Latvia	39.7	45.5	51.6	56.7	42.9
	Lebanon	51.8	59.8	68.6	74.9	44.7
	Lesotho	17.3	10.6	12	21	21.1
	Liberia	13	17.4	24.3	26.5	102.9
	Libya	48.2	52.3	61.5	60.2	24.9
	Lithuania	58.2	63.4	66	68.7	18.1
	Luxembourg	71.6	80	82.3	81.7	14.2
	Madagascar	21.7	19.3	22	24.9	14.5
	Malawi	18.8	18.2	24	28.3	50.1
	Malaysia	41.1	48.1	54.5	60.5	47.0
	Maldives	39.1	49	62	66.6	70.2
	Mali	14.8	19.1	21.6	25.6	73.5
	Malta	67.1	72.4	77.5	80.8	20.3
	Marshall Islands	33.5	34.4	38.8	43	28.5
	Mauritania	16.1	21.2	24.4	32.3	100.6
	Mauritius	51.3	59.3	60.8	63.7	24.3
	Mexico	31.2	43.5	50	55.4	77.3
	Micronesia (Federated States of)	32.8	38.8	43.2	47.6	45.1
	Monaco	79.7	82.4	85.3	86.4	8.4
	Mongolia	28.7	25.4	38.6	44.5	54.8
	Montenegro	75.4	74.6	75.5	79.4	5.4
	Morocco	26.8	34.1	39.9	47.6	77.9
	Mozambique	8.7	13.1	17.4	23.3	166.0
	Myanmar	29.2	34.4	40.5	49	67.7
	Namibia	18.8	18.6	27.5	38.3	103.8
	Nauru	49	49.2	48.8	58.4	19.1
	Nepal	18.1	27.5	34.2	40.9	126.1
	Netherlands	77.4	81.9	85.2	86.2	11.4
	New Zealand	74.2	74.8	77.1	78.9	6.4
	Nicaragua	36.5	31.3	43.8	50.4	38.2

Location type	Location name	QCI				% Change (1990 to 2019)
		1990	2000	2010	2019	
	Niger	10	11.6	16.5	19.7	96.9
	Nigeria	17.3	18.6	25.3	27.9	61.7
	Niue	53.8	61.2	65.6	67.1	24.6
	North Macedonia	60.8	64.7	68	70.5	15.9
	Northern Mariana Islands	63.5	71.3	69.4	69.9	10.2
	Norway	74.3	80.5	84	83.4	12.2
	Oman	42.9	55.1	57.5	58.9	37.5
	Pakistan	23	24.7	31.5	38.8	68.5
	Palau	57.3	63.4	64.2	66.2	15.6
	Palestine	34.7	34.5	35.2	44.6	28.6
	Panama	48.7	54.7	58.9	66	35.5
	Papua New Guinea	27.6	32.3	32.2	35.8	29.8
	Paraguay	42.7	45.6	49.8	58.5	37.2
	Peru	41.6	52.7	61	67.9	63.3
	Philippines	47.9	52.1	52.9	56.9	18.9
	Poland	30.3	39.2	44.7	47.9	58.0
	Portugal	74.1	84	87.4	88.4	19.3
	Puerto Rico	62.9	71.8	78.6	81.1	28.9
	Qatar	38	40	45.1	51.5	35.5
	Republic of Korea	71.7	76.5	86.7	89.7	25.1
	Republic of Moldova	49.4	57.6	61.4	66.7	35.1
	Romania	62.6	68.3	69.3	72.2	15.4
	Russian Federation	53.7	59.5	73	78	45.2
	Rwanda	12.1	10.4	22.8	31.1	155.7
	Saint Kitts and Nevis	55.2	58.9	57.9	58.3	5.6
	Saint Lucia	45.8	54.3	62.9	61.4	34.2
	Saint Vincent and the Grenadines	47.6	54	56	56.1	17.7
	Samoa	48.4	55.4	56.6	58.6	21.0
	San Marino	74	80.2	82.1	83.9	13.3
	Sao Tome and Principe	22.6	28.6	33.5	39	72.5
	Saudi Arabia	22.6	30.9	55.8	64.9	187.1
	Senegal	18	19	21.3	25.7	42.7
	Serbia	63.1	68.6	72.4	76	20.4
	Seychelles	50.1	54.3	59.8	62.8	25.4
	Sierra Leone	14.6	15.4	19.2	25.8	76.3
	Singapore	74.5	76.5	82.9	84.9	14.0
	Slovakia	76.4	79.1	83	86.3	13.0
	Slovenia	79.9	84.3	88.4	90.8	13.7
	Solomon Islands	38	44.8	45.3	50.3	32.5
	Somalia	7.7	3.4	4.3	7.6	-1.6
	South Africa	44.8	33	35.7	45.6	1.7
	South Sudan	12	9.7	12.2	14.3	19.8
	Spain	86.7	90.5	92.7	93.2	7.5
	Sri Lanka	49.2	52.4	60	67.3	36.7
	Sudan	23.3	29.8	34.9	42.7	83.4
	Suriname	38.4	42.9	48.2	53.1	38.3
	Sweden	73.3	74.1	75	77.4	5.5
	Switzerland	72.7	78.5	79.7	79.7	9.6

Location type	Location name	QCI				% Change (1990 to 2019)
		1990	2000	2010	2019	
	Syrian Arab Republic	42.5	48	52.1	55	29.2
	Taiwan (Province of China)	67.2	73	77.4	79.4	18.2
	Tajikistan	41.2	38.1	35.8	39.7	-3.6
	Thailand	50.4	61.5	66.9	72.3	43.5
	Timor-Leste	20.6	27	34.8	42.4	105.3
	Togo	22.9	19.2	22.9	29.3	28.3
	Tokelau	42.4	50.1	56.1	60.9	43.5
	Tonga	42.6	47.7	47	50.2	17.7
	Trinidad and Tobago	38.9	44.4	54.6	55.4	42.2
	Tunisia	49.8	57.4	62	67.6	35.7
	Turkey	35.7	45.8	54.2	57.2	60.3
	Turkmenistan	47	50.1	55.7	61	29.9
	Tuvalu	38.3	45.6	48.6	52	35.7
	Uganda	15.7	18	23.6	30.1	91.1
	Ukraine	51.7	54	62.3	65.7	27.2
	United Arab Emirates	22.3	24.8	19.9	41.1	84.3
	United Kingdom	71	74.3	80	80.6	13.5
	United Republic of Tanzania	19.7	19.5	24.5	30.1	52.6
	United States Virgin Islands	49.1	55.2	59.4	59.2	20.6
	United States of America	77.5	81.2	80.4	80.5	3.8
	Uruguay	65	68.2	71.8	74.8	15.2
	Uzbekistan	50.5	50.6	52.3	57.7	14.2
	Vanuatu	29.3	37	35.7	38.4	30.9
	Venezuela (Bolivarian Republic of)	41	52.6	62.5	65.3	59.3
	Viet Nam	39.2	45.6	53.1	60.8	55.3
	Yemen	21.8	26.1	33	36.4	67.5
	Zambia	17.5	14.5	23.2	34.5	97.2
	Zimbabwe	30.3	37.9	21.3	27.5	-9.2
