# Supplementary material for: Global, regional, and national quality of care index of cervical and ovarian cancer: a systematic analysis for the global burden of disease study 1990–2019
Source: BMC Womens Health. 2024 Jan 25;24:69. doi: 10.1186/s12905-024-02884-9 (PMC10809627; doi:10.1186/s12905-024-02884-9)
Supplement: Supplementary file 3 — Additional file 3: Supplementary Table 3. The QCI for cervical cancer from 1990 to 2019 among different age groups. [file 12905_2024_2884_MOESM3_ESM.pdf]

Age group	Year			
	1990	2000	2010	2019
15-49 years	49.0	53.3	60.0	63.5
15 to 19	47.6	53.1	60.5	64.6
20 to 24	48.1	52.8	60.2	63.4
25 to 29	50.0	53.6	60.8	64.5
30 to 34	52.7	56.3	63.0	67.5
35 to 39	51.4	56.1	63.1	66.5
40 to 44	49.4	54.9	62.5	65.3
45 to 49	45.1	51.1	59.5	63.2
50-69 years	41.5	45.5	52.7	56.2
50 to 54	43.8	49.2	56.7	60.6
55 to 59	42.1	46.4	53.5	57.3
60 to 64	41.4	44.2	50.2	53.7
65 to 69	39.5	41.2	45.2	48.5
70+ years	34.0	37.0	39.2	40.3
70 to 74	34.4	37.7	41.0	43.2
75 to 79	34.0	37.2	39.5	41.4
80+ years	36.8	37.7	41.5	40.6
Age-standardized	43.1	48.3	54.9	58.5
