# Supplementary material for: Global, regional, and national quality of care index of cervical and ovarian cancer: a systematic analysis for the global burden of disease study 1990–2019
Source: BMC Womens Health. 2024 Jan 25;24:69. doi: 10.1186/s12905-024-02884-9 (PMC10809627; doi:10.1186/s12905-024-02884-9)
Supplement: Supplementary file 4 — Additional file 4: Supplementary Table 4. All ages and age-standardized burden of ovarian cancer from 1990 to 2019 in different locations. [file 12905_2024_2884_MOESM4_ESM.pdf]

Location type	Location name	Measure	Age, Metric	Burden 1990	Burden 2000	Burden 2010	Burden 2019	% Change (1990 to 2019)
**Global**		**Incidence**	All ages (number)	141706 (130541 to 160779)	182796 (171178 to 200661)	229929 (214203 to 247838)	294422 (260649 to 329727)	107.8 (76.1 to 135.7)
			Age-standardized rate (per 100,000)	6.5 (6 to 7.3)	6.7 (6.3 to 7.3)	6.6 (6.2 to 7.1)	6.9 (6.1 to 7.7)	6.3 (-10.1 to 20.4)
		**Prevalence**	All ages (number)	561475 (514350 to 641525)	746357 (691108 to 821350)	950867 (888453 to 1023111)	1206652 (1064857 to 1353763)	114.9 (80.7 to 145)
			Age-standardized rate (per 100,000)	24.7 (22.8 to 28.1)	26.6 (24.8 to 29.2)	27.1 (25.3 to 29.1)	28.6 (25.2 to 32.1)	15.4 (-2.6 to 31.5)
		**Deaths**	All ages (number)	97363 (89703 to 109761)	122621 (115063 to 133712)	153456 (141768 to 164591)	198412 (175357 to 217665)	103.8 (75.7 to 126.4)
			Age-standardized rate (per 100,000)	4.6 (4.2 to 5.2)	4.6 (4.4 to 5)	4.5 (4.2 to 4.8)	4.6 (4 to 5)	-0.5 (-14 to 10.5)
		**DALYs**	All ages (number)	2732666 (2493732 to 3165170)	3400481 (3169502 to 3790725)	4204876 (3884973 to 4545902)	5359737 (4692949 to 5954993)	96.1 (65 to 120.5)
			Age-standardized rate (per 100,000)	124.1 (113.7 to 143)	124.6 (116.6 to 138.4)	121.2 (112.1 to 130.9)	124.7 (109.1 to 138.7)	0.5 (-15.4 to 12.9)
		**YLLs**	All ages (number)	2659975 (2425940 to 3089596)	3305225 (3080147 to 3686841)	4083787 (3796904 to 4411059)	5205660 (4579409 to 5768211)	95.7 (64.5 to 120.3)
			Age-standardized rate (per 100,000)	120.8 (110.6 to 139.5)	121.2 (113.4 to 134.9)	117.7 (109.5 to 127.2)	121.1 (106.4 to 134.3)	0.2 (-15.6 to 12.8)
		**YLDs**	All ages (number)	72691 (51949 to 94049)	95256 (68792 to 122722)	121089 (87851 to 155255)	154077 (111432 to 199195)	112 (80.8 to 140.7)
			Age-standardized rate (per 100,000)	3.3 (2.4 to 4.3)	3.5 (2.5 to 4.5)	3.5 (2.5 to 4.5)	3.6 (2.6 to 4.7)	9.5 (-7 to 24.4)
**World Bank Income Levels**	**World Bank High Income**	**Incidence**	All ages (number)	75795 (69598 to 78068)	87078 (82381 to 89223)	91235 (85368 to 95050)	98039 (86477 to 111022)	29.3 (15 to 49.6)
			Age-standardized rate (per 100,000)	11.4 (10.4 to 11.7)	11.3 (10.8 to 11.5)	10.1 (9.6 to 10.5)	9.5 (8.4 to 10.8)	-16.6 (-26.2 to -2.5)
		**Prevalence**	All ages (number)	300182 (274076 to 308690)	352523 (340196 to 359890)	370326 (354077 to 385448)	387744 (343583 to 440120)	29.2 (14.1 to 51.9)
			Age-standardized rate (per 100,000)	48.4 (44.1 to 49.7)	49.9 (48.5 to 50.9)	45.6 (43.9 to 47.5)	43.2 (38.3 to 49.1)	-10.9 (-21.6 to 6.1)
		**Deaths**	All ages (number)	52539 (48247 to 54310)	59663 (55440 to 61562)	63052 (57540 to 66058)	69467 (61668 to 75181)	32.2 (20.9 to 47.1)
			Age-standardized rate (per 100,000)	7.4 (6.7 to 7.6)	7 (6.6 to 7.2)	6.2 (5.7 to 6.4)	5.8 (5.3 to 6.2)	-21.3 (-27.7 to -10)
		**DALYs**	All ages (number)	1297720 (1188195 to 1336622)	1403747 (1338527 to 1437558)	1424225 (1346732 to 1481329)	1510884 (1390475 to 1628908)	16.4 (7 to 35.3)
			Age-standardized rate (per 100,000)	198 (180.9 to 203.7)	184.6 (177.9 to 188.9)	159.1 (151.8 to 165)	148 (137 to 159.8)	-25.2 (-31.2 to -11.6)

Location type	Location name	Measure	Age, Metric	Burden				% Change (1990 to 2019)
				1990	2000	2010	2019	
		YLLs	All ages (number)	1258002 (1152019 to 1292778)	1357229 (1297162 to 1386797)	1375210 (1299729 to 1431629)	1458756 (1342419 to 1570417)	16 (6.5 to 34.8)
			Age-standardized rate (per 100,000)	191.9 (175.1 to 197.1)	178.4 (171.1 to 181.9)	153.4 (146.6 to 159.1)	142.7 (132.3 to 154.1)	-25.6 (-31.5 to -12)
		YLDs	All ages (number)	39718 (28801 to 50451)	46518 (33765 to 59231)	49015 (35625 to 62593)	52128 (37358 to 68078)	31.2 (16.1 to 53)
			Age-standardized rate (per 100,000)	6.1 (4.4 to 7.8)	6.2 (4.5 to 7.9)	5.6 (4.1 to 7.2)	5.3 (3.8 to 7)	-13.1 (-23.7 to 2.5)
	World Bank Upper Middle Income	Incidence	All ages (number)	39981 (36213 to 45863)	56678 (52652 to 63296)	77990 (72318 to 85844)	101597 (85089 to 116382)	154.1 (104.1 to 198.6)
			Age-standardized rate (per 100,000)	4.5 (4.2 to 5.2)	5.1 (4.7 to 5.7)	5.5 (5.1 to 6.1)	5.9 (4.9 to 6.7)	29.2 (4 to 51.7)
		Prevalence	All ages (number)	162093 (142864 to 187021)	238937 (215719 to 262374)	333488 (308021 to 367145)	430342 (359553 to 495298)	165.5 (110.1 to 214.6)
			Age-standardized rate (per 100,000)	17.5 (15.5 to 20.1)	20.5 (18.7 to 22.5)	23.2 (21.4 to 25.5)	25.8 (21.5 to 29.6)	47.4 (16.9 to 74.9)
		Deaths	All ages (number)	26805 (24591 to 30845)	36677 (34576 to 41406)	50152 (45886 to 54640)	66258 (55420 to 75367)	147.2 (98.6 to 189.4)
			Age-standardized rate (per 100,000)	3.2 (3 to 3.7)	3.4 (3.2 to 3.9)	3.6 (3.3 to 3.9)	3.7 (3.1 to 4.2)	14.8 (-7.7 to 34.1)
		DALYs	All ages (number)	844559 (766004 to 973776)	1141370 (1069533 to 1273072)	1487328 (1364854 to 1623633)	1881410 (1573261 to 2142081)	122.8 (77.8 to 161.9)
			Age-standardized rate (per 100,000)	95.9 (87.3 to 110.2)	102.1 (96.2 to 114)	104.1 (95.5 to 113.6)	106.4 (89.1 to 120.9)	10.9 (-11.5 to 30.2)
		YLLs	All ages (number)	824434 (743887 to 951172)	1112126 (1043995 to 1237775)	1445888 (1326818 to 1573055)	1827273 (1528614 to 2086965)	121.6 (77.2 to 161.1)
			Age-standardized rate (per 100,000)	93.7 (85.1 to 108)	99.5 (93.7 to 111.4)	101.2 (93 to 110)	103.3 (86.6 to 117.8)	10.2 (-12 to 29.7)
		YLDs	All ages (number)	20125 (14352 to 26535)	29244 (21016 to 37764)	41440 (29758 to 54280)	54137 (38022 to 72221)	169 (114.4 to 220.2)
			Age-standardized rate (per 100,000)	2.3 (1.6 to 3)	2.6 (1.9 to 3.3)	2.9 (2.1 to 3.8)	3.1 (2.2 to 4.2)	38 (9.9 to 63.3)
	World Bank Lower Middle Income	Incidence	All ages (number)	23005 (18259 to 32924)	34931 (29471 to 45372)	54533 (46069 to 65156)	84862 (68602 to 103948)	268.9 (136.1 to 370.5)
			Age-standardized rate (per 100,000)	3.8 (3.1 to 5.3)	4.5 (3.8 to 5.7)	5.2 (4.5 to 6.2)	6.3 (5.1 to 7.6)	65 (8.7 to 108.1)
		Prevalence	All ages (number)	88772 (68334 to 128920)	139945 (115371 to 182610)	223793 (186364 to 270048)	349866 (279214 to 430314)	294.1 (145.7 to 406.9)
			Age-standardized rate (per 100,000)	13.2 (10.4 to 18.7)	16.1 (13.5 to 20.8)	19.6 (16.4 to 23.4)	24.2 (19.4 to 29.7)	83.6 (17.6 to 134.3)

Location type	Location name	Measure	Age, Metric	Burden				% Change (1990 to 2019)
				1990	2000	2010	2019	
		Deaths	All ages (number)	15893 (12898 to 22356)	23385 (20184 to 30040)	35932 (30940 to 42722)	55844 (44897 to 69102)	251.4 (131.5 to 340.7)
			Age-standardized rate (per 100,000)	2.9 (2.4 to 4)	3.3 (2.9 to 4.2)	3.7 (3.2 to 4.4)	4.4 (3.6 to 5.4)	53.1 (2.2 to 91.3)
		DALYs	All ages (number)	519883 (409803 to 751569)	760077 (644144 to 999410)	1152511 (974866 to 1373121)	1744032 (1379802 to 2164085)	235.5 (115.2 to 326.3)
			Age-standardized rate (per 100,000)	83.9 (67.4 to 119.1)	95.9 (82.4 to 124.4)	109 (92.6 to 130)	127.2 (101 to 157.6)	51.6 (-0.8 to 90.9)
		YLLs	All ages (number)	508457 (400176 to 738312)	742600 (628792 to 977745)	1124906 (950184 to 1344918)	1701093 (1346144 to 2117388)	234.6 (114.7 to 325.5)
			Age-standardized rate (per 100,000)	82.1 (65.8 to 116.9)	93.7 (80.4 to 121.7)	106.4 (90.6 to 126.7)	124.1 (98.6 to 154.2)	51.2 (-0.9 to 90.9)
		YLDs	All ages (number)	11427 (7735 to 17066)	17478 (12187 to 24289)	27605 (19227 to 36860)	42939 (29188 to 59162)	275.8 (140.2 to 379)
			Age-standardized rate (per 100,000)	1.8 (1.2 to 2.7)	2.2 (1.5 to 3)	2.6 (1.8 to 3.4)	3.1 (2.1 to 4.3)	68 (10 to 113.2)
	World Bank Low Income	Incidence	All ages (number)	2866 (1910 to 6317)	4014 (2925 to 7768)	6045 (4732 to 9523)	9771 (7806 to 13296)	240.9 (99.5 to 418.4)
			Age-standardized rate (per 100,000)	3.1 (2.1 to 6.6)	3.5 (2.6 to 6.5)	4 (3.2 to 6.2)	4.9 (4 to 6.6)	55.9 (-6.6 to 132.8)
		Prevalence	All ages (number)	10194 (6735 to 23095)	14555 (10538 to 28979)	22733 (17412 to 36325)	38052 (29857 to 52645)	273.3 (112.3 to 485.5)
			Age-standardized rate (per 100,000)	9.8 (6.5 to 21.6)	11 (8 to 21.2)	13.2 (10.3 to 20.6)	16.6 (13.2 to 22.8)	69.5 (-1.1 to 158.5)
		Deaths	All ages (number)	2087 (1398 to 4483)	2834 (2093 to 5379)	4238 (3355 to 6575)	6742 (5475 to 8987)	223.1 (89.6 to 375.1)
			Age-standardized rate (per 100,000)	2.5 (1.7 to 5.2)	2.7 (2 to 5)	3.2 (2.5 to 4.7)	3.8 (3.1 to 4.9)	50.2 (-9.9 to 118.3)
		DALYs	All ages (number)	69396 (45830 to 157072)	93539 (67689 to 186589)	138536 (108481 to 224194)	220627 (175376 to 301015)	217.9 (81.4 to 382.5)
			Age-standardized rate (per 100,000)	73.5 (49.1 to 161.2)	78.9 (57.7 to 153.1)	90.4 (71.3 to 142.8)	108.4 (87.2 to 145.7)	47.4 (-14.4 to 118.8)
		YLLs	All ages (number)	68005 (44759 to 154308)	91572 (66310 to 182432)	135573 (106373 to 218990)	215834 (172054 to 293370)	217.4 (81.3 to 381.5)
			Age-standardized rate (per 100,000)	72.1 (47.9 to 158.2)	77.3 (56.4 to 149.9)	88.5 (69.8 to 139.5)	106.1 (85.4 to 142.4)	47.2 (-14.5 to 118.4)
		YLDs	All ages (number)	1392 (801 to 3028)	1967 (1221 to 3821)	2963 (1925 to 4690)	4793 (3194 to 7071)	244.4 (97.6 to 428.7)
			Age-standardized rate (per 100,000)	1.5 (0.9 to 3.2)	1.7 (1 to 3.1)	1.9 (1.2 to 3)	2.3 (1.6 to 3.4)	56.6 (-8 to 133.8)

Location type	Location name	Measure	Age, Metric	Burden				% Change (1990 to 2019)
				1990	2000	2010	2019	
SDI	High SDI	Incidence	All ages (number)	62463 (56715 to 64466)	71263 (67407 to 73109)	74193 (69336 to 77405)	80454 (70504 to 91461)	28.8 (13.9 to 49.8)
			Age-standardized rate (per 100,000)	11.5 (10.4 to 11.8)	11.2 (10.7 to 11.5)	9.9 (9.4 to 10.3)	9.3 (8.2 to 10.6)	-18.8 (-28.6 to -3.8)
		Prevalence	All ages (number)	246101 (223063 to 253363)	289008 (278263 to 295068)	302862 (289097 to 315583)	319760 (283598 to 361982)	29.9 (14.2 to 54.4)
			Age-standardized rate (per 100,000)	48.4 (43.8 to 49.8)	49.6 (48.1 to 50.6)	44.9 (43 to 46.9)	42.3 (37.4 to 48.1)	-12.6 (-23.2 to 5.1)
		Deaths	All ages (number)	43458 (39182 to 45018)	48682 (45166 to 50292)	50935 (46505 to 53406)	56639 (50391 to 61318)	30.3 (19.1 to 47.3)
			Age-standardized rate (per 100,000)	7.5 (6.7 to 7.7)	7 (6.6 to 7.2)	6 (5.6 to 6.3)	5.7 (5.2 to 6.1)	-24 (-30.3 to -12.3)
		DALYs	All ages (number)	1061103 (956786 to 1094307)	1137377 (1084170 to 1167188)	1146048 (1083722 to 1191979)	1229123 (1125703 to 1323417)	15.8 (6.5 to 35.4)
			Age-standardized rate (per 100,000)	198.3 (178.8 to 204.3)	182.1 (174.9 to 186.6)	154.6 (147.5 to 160.8)	143.8 (132.6 to 154.5)	-27.5 (-33.1 to -14.2)
		YLLs	All ages (number)	1028371 (929308 to 1059283)	1099193 (1044775 to 1124407)	1105990 (1045267 to 1150839)	1186172 (1088737 to 1276255)	15.3 (5.9 to 34.9)
			Age-standardized rate (per 100,000)	192.2 (173.6 to 197.7)	175.9 (168.8 to 179.6)	149.1 (142.3 to 154.7)	138.6 (128.3 to 149.2)	-27.9 (-33.6 to -14.7)
		YLDs	All ages (number)	32732 (23791 to 41748)	38184 (27622 to 48642)	40058 (29085 to 51386)	42952 (30604 to 56621)	31.2 (15.6 to 54.7)
			Age-standardized rate (per 100,000)	6.1 (4.5 to 7.9)	6.2 (4.5 to 7.9)	5.5 (4 to 7.1)	5.2 (3.7 to 6.9)	-15.3 (-25.7 to 1.3)
	High-middle SDI	Incidence	All ages (number)	43567 (40298 to 46702)	54530 (51297 to 57237)	66408 (61875 to 69950)	77286 (65885 to 86459)	77.4 (54.3 to 100)
			Age-standardized rate (per 100,000)	7.3 (6.7 to 7.8)	7.7 (7.2 to 8)	7.7 (7.1 to 8.1)	7.6 (6.4 to 8.5)	3.4 (-9.8 to 16.4)
		Prevalence	All ages (number)	174035 (158421 to 187558)	221446 (204847 to 233414)	275192 (256206 to 290232)	319348 (270469 to 358032)	83.5 (59.5 to 106.9)
			Age-standardized rate (per 100,000)	29.4 (26.8 to 31.7)	31.4 (29 to 33.1)	32.6 (30.3 to 34.4)	33.3 (28.2 to 37.3)	13.1 (-2 to 28)
		Deaths	All ages (number)	29784 (27764 to 31762)	36850 (35025 to 38431)	44369 (41253 to 46550)	51967 (44998 to 57246)	74.5 (52.6 to 92.9)
			Age-standardized rate (per 100,000)	5 (4.6 to 5.3)	5.1 (4.9 to 5.3)	5 (4.6 to 5.2)	4.7 (4.1 to 5.2)	-4.5 (-16.4 to 5.7)
		DALYs	All ages (number)	864811 (793572 to 925109)	1042139 (979751 to 1087989)	1213384 (1118107 to 1271329)	1378231 (1191048 to 1526401)	59.4 (38.7 to 76.9)
			Age-standardized rate (per 100,000)	145.1 (132.9 to 155.3)	146.4 (137.3 to 152.9)	139.4 (128.5 to 146.1)	133 (114.8 to 147.5)	-8.3 (-20.1 to 1.9)

Location type	Location name	Measure	Age, Metric	Burden				% Change
				1990	2000	2010	2019	(1990 to 2019)
		YLLs	All ages (number)	842608 (772891 to 901624)	1013974 (954015 to 1057826)	1178368 (1087868 to 1233928)	1337300 (1154016 to 1480447)	58.7 (37.7 to 76.4)
			Age-standardized rate (per 100,000)	141.3 (129.4 to 151.3)	142.5 (133.8 to 148.6)	135.4 (124.9 to 141.8)	129 (111.4 to 142.9)	-8.7 (-20.8 to 1.5)
		YLDs	All ages (number)	22204 (15994 to 28732)	28165 (20577 to 36273)	35016 (25508 to 44952)	40931 (29187 to 53714)	84.3 (59.7 to 110.7)
			Age-standardized rate (per 100,000)	3.7 (2.7 to 4.8)	4 (2.9 to 5.1)	4.1 (3 to 5.2)	4.1 (2.9 to 5.3)	8.5 (-6 to 24.2)
	Middle SDI	Incidence	All ages (number)	21289 (18298 to 27014)	34518 (30451 to 40138)	52568 (46976 to 60491)	76545 (63249 to 88974)	259.6 (152.7 to 331.7)
			Age-standardized rate (per 100,000)	3.4 (3 to 4.3)	4.2 (3.8 to 4.9)	4.9 (4.4 to 5.7)	5.7 (4.7 to 6.6)	67 (18.7 to 100.6)
		Prevalence	All ages (number)	89213 (74272 to 114785)	150915 (128692 to 178211)	230351 (206426 to 265101)	330955 (273729 to 386575)	271 (157.1 to 348.6)
			Age-standardized rate (per 100,000)	12.9 (11 to 16.4)	17 (14.8 to 19.8)	20.5 (18.3 to 23.5)	24.5 (20.2 to 28.5)	89.4 (32.5 to 127.5)
		Deaths	All ages (number)	13706 (12089 to 17148)	21357 (19487 to 25180)	32694 (29063 to 37043)	48485 (39894 to 56526)	253.8 (155.6 to 324.8)
			Age-standardized rate (per 100,000)	2.4 (2.2 to 3)	2.9 (2.6 to 3.4)	3.3 (2.9 to 3.7)	3.7 (3 to 4.3)	49.9 (9.2 to 79.4)
		DALYs	All ages (number)	462013 (401880 to 592519)	703917 (631766 to 819555)	1026825 (908838 to 1167372)	1453634 (1199319 to 1696724)	214.6 (124 to 277.4)
			Age-standardized rate (per 100,000)	73.9 (64.7 to 93.5)	86.1 (78.3 to 100.8)	95.5 (84.5 to 108.7)	106.4 (87.7 to 124)	44 (3.5 to 73.5)
		YLLs	All ages (number)	451249 (391843 to 578539)	686016 (618597 to 798686)	998925 (883942 to 1137613)	1413034 (1163156 to 1651798)	213.1 (123.1 to 276.5)
			Age-standardized rate (per 100,000)	72.2 (63.1 to 91.1)	84 (76.2 to 98.6)	92.9 (82.2 to 105.7)	103.4 (85.2 to 120.8)	43.3 (3 to 72.9)
		YLDs	All ages (number)	10764 (7390 to 14970)	17901 (12664 to 23573)	27901 (19969 to 36875)	40599 (28081 to 54222)	277.2 (164.9 to 355.1)
			Age-standardized rate (per 100,000)	1.7 (1.2 to 2.3)	2.1 (1.5 to 2.8)	2.6 (1.8 to 3.4)	3 (2.1 to 4)	76.1 (23.8 to 112.4)
	Low-middle SDI	Incidence	All ages (number)	10234 (8031 to 16701)	16454 (13808 to 23697)	26984 (23303 to 33045)	43595 (35303 to 54683)	326 (158.8 to 470.5)
			Age-standardized rate (per 100,000)	3 (2.3 to 4.7)	3.7 (3.1 to 5.2)	4.5 (3.9 to 5.5)	5.6 (4.6 to 7.1)	91.3 (17.8 to 157.1)
		Prevalence	All ages (number)	37821 (29178 to 63923)	63369 (52867 to 93473)	105837 (90716 to 131131)	172394 (138748 to 216999)	355.8 (166.8 to 514.4)
			Age-standardized rate (per 100,000)	9.6 (7.5 to 15.7)	12.6 (10.5 to 18.2)	16.2 (13.9 to 20)	21.1 (17 to 26.6)	119 (31.2 to 195.1)

Location type	Location name	Measure	Age, Metric	Burden				% Change (1990 to 2019)
				1990	2000	2010	2019	
		Deaths	All ages (number)	7311 (5765 to 11582)	11352 (9553 to 15989)	18541 (16106 to 22445)	29874 (24421 to 37624)	308.6 (151.3 to 445.8)
			Age-standardized rate (per 100,000)	2.3 (1.8 to 3.6)	2.8 (2.4 to 3.8)	3.4 (2.9 to 4.1)	4.1 (3.4 to 5.1)	75.1 (10.1 to 134.4)
		DALYs	All ages (number)	241438 (188017 to 394770)	371082 (311125 to 534788)	589765 (511642 to 714285)	922653 (740013 to 1169375)	282.1 (129.6 to 412.9)
			Age-standardized rate (per 100,000)	67.6 (53.3 to 107.7)	81 (68.1 to 115.7)	97.4 (84.6 to 117.4)	118.4 (95.4 to 150.2)	75.1 (6.9 to 134.6)
		YLLs	All ages (number)	236426 (183743 to 386877)	362974 (304016 to 524902)	576387 (499422 to 696895)	901082 (721249 to 1144629)	281.1 (128.6 to 412.2)
			Age-standardized rate (per 100,000)	66.2 (52 to 105.7)	79.2 (66.6 to 113)	95.2 (82.7 to 114.7)	115.7 (93.1 to 146.6)	74.7 (6.5 to 134.1)
		YLDs	All ages (number)	5012 (3270 to 8440)	8108 (5489 to 12086)	13378 (9229 to 17951)	21572 (14904 to 29906)	330.4 (159.3 to 479.2)
			Age-standardized rate (per 100,000)	1.4 (0.9 to 2.3)	1.8 (1.2 to 2.6)	2.2 (1.5 to 2.9)	2.7 (1.9 to 3.8)	94.4 (19.5 to 162.1)
	Low SDI	Incidence	All ages (number)	4095 (2808 to 8460)	5937 (4470 to 10947)	9651 (7746 to 14268)	16389 (13486 to 20299)	300.2 (126.2 to 485.4)
			Age-standardized rate (per 100,000)	3 (2 to 5.9)	3.4 (2.6 to 6)	4.1 (3.3 to 5.9)	5.1 (4.3 to 6.3)	73.9 (1.8 to 149.2)
		Prevalence	All ages (number)	14072 (9569 to 29891)	21224 (15937 to 40010)	36101 (28986 to 53986)	63551 (52122 to 78690)	351.6 (145.6 to 575.9)
			Age-standardized rate (per 100,000)	8.9 (6.1 to 18.2)	10.4 (7.8 to 19.1)	13.2 (10.6 to 19.5)	17.4 (14.3 to 21.5)	95.8 (10.3 to 188.7)
		Deaths	All ages (number)	3065 (2119 to 6167)	4318 (3188 to 7747)	6835 (5482 to 9995)	11346 (9551 to 13928)	270.2 (115.2 to 433.2)
			Age-standardized rate (per 100,000)	2.4 (1.7 to 4.7)	2.7 (2 to 4.7)	3.3 (2.6 to 4.7)	4 (3.4 to 4.9)	63.8 (-2.1 to 134)
		DALYs	All ages (number)	102198 (69309 to 215443)	144224 (105294 to 270072)	226586 (181036 to 341988)	373324 (311090 to 462226)	265.3 (104.7 to 436.5)
			Age-standardized rate (per 100,000)	71 (48.9 to 144.9)	79.3 (58.2 to 144.3)	93.8 (75.4 to 138.5)	115.2 (96.4 to 141.8)	62.1 (-6.4 to 134.4)
		YLLs	All ages (number)	100249 (68031 to 211580)	141376 (103146 to 264335)	221916 (176977 to 333083)	365380 (303521 to 453302)	264.5 (104.3 to 435)
			Age-standardized rate (per 100,000)	69.7 (48 to 142.2)	77.8 (57.2 to 141.3)	91.9 (73.8 to 135.9)	112.8 (94.1 to 139.4)	61.9 (-6.8 to 134)
		YLDs	All ages (number)	1949 (1149 to 3910)	2848 (1806 to 5189)	4670 (3087 to 6972)	7943 (5519 to 10942)	307.5 (128.6 to 503.2)
			Age-standardized rate (per 100,000)	1.4 (0.8 to 2.6)	1.6 (1 to 2.7)	1.9 (1.3 to 2.8)	2.4 (1.7 to 3.3)	75.8 (2.1 to 157.9)

Location type	Location name	Measure	Age, Metric	Burden 1990	Burden 2000	Burden 2010	Burden 2019	% Change (1990 to 2019)
WHO Regions	African Region	Incidence	All ages (number)	4196 (3119 to 7269)	6229 (4935 to 9183)	9478 (7930 to 11932)	15412 (12765 to 18662)	267.3 (110.4 to 417.5)
			Age-standardized rate (per 100,000)	3.2 (2.3 to 5.3)	3.6 (2.9 to 5.2)	4.2 (3.5 to 5.2)	4.9 (4.1 to 5.9)	56.8 (-7.5 to 114.1)
		Prevalence	All ages (number)	14844 (10961 to 26151)	22580 (17799 to 33437)	35537 (29552 to 44944)	60076 (49576 to 74290)	304.7 (128.5 to 481.7)
			Age-standardized rate (per 100,000)	9.7 (7.3 to 16.7)	11.3 (9 to 16.4)	13.3 (11.2 to 16.6)	16.6 (13.8 to 20.2)	70.8 (-1.2 to 139.7)
		Deaths	All ages (number)	3089 (2296 to 5235)	4434 (3524 to 6534)	6718 (5605 to 8445)	10710 (8854 to 12748)	246.8 (99.7 to 377.2)
			Age-standardized rate (per 100,000)	2.6 (1.9 to 4.2)	2.9 (2.3 to 4.2)	3.3 (2.8 to 4.1)	3.9 (3.2 to 4.6)	51.5 (-10.4 to 104.5)
		DALYs	All ages (number)	100089 (73801 to 178068)	143480 (112569 to 219493)	216230 (178147 to 278104)	345283 (283387 to 419122)	245 (91 to 389)
			Age-standardized rate (per 100,000)	73.2 (54.2 to 127.3)	81.7 (64.8 to 122.2)	92.9 (77.3 to 117.5)	108.4 (89.5 to 130.4)	48.2 (-16.1 to 105.6)
		YLLs	All ages (number)	98073 (72212 to 175121)	140458 (110337 to 215127)	211597 (174834 to 272456)	337722 (277835 to 410785)	244.4 (90.8 to 388.5)
			Age-standardized rate (per 100,000)	71.7 (52.9 to 124.7)	80 (63.4 to 119.8)	91 (75.9 to 115.1)	106.1 (87.6 to 127.1)	48 (-16.3 to 105.4)
		YLDs	All ages (number)	2016 (1268 to 3423)	3023 (1992 to 4595)	4633 (3158 to 6372)	7561 (5188 to 10534)	275 (115.2 to 433)
			Age-standardized rate (per 100,000)	1.5 (0.9 to 2.5)	1.7 (1.1 to 2.5)	2 (1.3 to 2.7)	2.3 (1.6 to 3.2)	58 (-7.4 to 119.6)
	Eastern Mediterranean Region	Incidence	All ages (number)	3790 (2938 to 5903)	6540 (5572 to 8553)	11774 (9426 to 14265)	19838 (13825 to 26991)	423.4 (155.5 to 734.6)
			Age-standardized rate (per 100,000)	3.7 (2.8 to 5.7)	4.8 (4.2 to 6.2)	6.3 (5.1 to 7.7)	7.8 (5.6 to 10.6)	111.5 (5 to 237.2)
		Prevalence	All ages (number)	14254 (10781 to 22579)	26089 (21394 to 34325)	49196 (38273 to 59640)	85881 (58558 to 118093)	502.5 (188.6 to 852.5)
			Age-standardized rate (per 100,000)	12.2 (9.4 to 19.1)	16.7 (14.1 to 21.8)	22.8 (18.2 to 27.5)	29.9 (20.9 to 40.6)	144.4 (19.3 to 287.3)
		Deaths	All ages (number)	2691 (2069 to 4139)	4419 (3816 to 5778)	7590 (6147 to 9260)	12381 (8675 to 17228)	360 (127.4 to 632.4)
			Age-standardized rate (per 100,000)	2.9 (2.2 to 4.4)	3.6 (3.2 to 4.7)	4.6 (3.8 to 5.8)	5.5 (3.9 to 7.8)	90.3 (-5.4 to 203.3)
		DALYs	All ages (number)	89008 (68768 to 140518)	148524 (121427 to 197665)	256591 (200587 to 312820)	417061 (283568 to 581659)	368.6 (127.7 to 645.2)
			Age-standardized rate (per 100,000)	84.7 (65.5 to 131.9)	107.2 (91.1 to 141.2)	134.9 (108.3 to 164.5)	161.8 (111.9 to 225)	91.1 (-6.2 to 202.6)

Location type	Location name	Measure	Age, Metric	Burden 1990	Burden 2000	Burden 2010	Burden 2019	% Change (1990 to 2019)
		YLLs	All ages (number)	87176 (67448 to 137588)	145305 (118991 to 193254)	250708 (195421 to 306504)	407000 (276999 to 568923)	366.9 (127.1 to 644.2)
			Age-standardized rate (per 100,000)	82.9 (64.1 to 129.3)	104.9 (89.2 to 137.9)	131.9 (105.5 to 161.2)	158 (109.2 to 220.4)	90.5 (-6.5 to 204.3)
		YLDs	All ages (number)	1832 (1198 to 2950)	3219 (2237 to 4455)	5883 (3855 to 8118)	10061 (6132 to 15232)	449.3 (169.6 to 771.5)
			Age-standardized rate (per 100,000)	1.7 (1.1 to 2.7)	2.3 (1.6 to 3.1)	3 (2 to 4.1)	3.8 (2.4 to 5.7)	119.9 (9.2 to 251.4)
	European Region	Incidence	All ages (number)	64408 (57705 to 66790)	71042 (67906 to 72844)	75234 (71470 to 77512)	79091 (69855 to 89199)	22.8 (10.3 to 44.5)
			Age-standardized rate (per 100,000)	11.2 (10 to 11.7)	11.3 (10.9 to 11.6)	10.7 (10.3 to 11)	10.3 (9.1 to 11.6)	-8.4 (-17.8 to 8.5)
		Prevalence	All ages (number)	249608 (218191 to 258694)	278235 (268806 to 284817)	296090 (285433 to 304602)	308328 (273659 to 349415)	23.5 (10.2 to 47.4)
			Age-standardized rate (per 100,000)	46.9 (40.5 to 48.7)	48 (46.5 to 49.2)	46.6 (45.2 to 47.9)	45.3 (40 to 51.5)	-3.3 (-13.9 to 16.6)
		Deaths	All ages (number)	45432 (41488 to 47136)	49945 (47180 to 51366)	53121 (49344 to 54993)	56568 (50623 to 61385)	24.5 (14.2 to 39.2)
			Age-standardized rate (per 100,000)	7.4 (6.7 to 7.7)	7.3 (7 to 7.5)	6.9 (6.5 to 7.1)	6.5 (5.8 to 7)	-12.4 (-19.2 to 0.7)
		DALYs	All ages (number)	1180593 (1057363 to 1225288)	1246311 (1198402 to 1278231)	1278107 (1224407 to 1315339)	1316591 (1184739 to 1432571)	11.5 (3 to 31.1)
			Age-standardized rate (per 100,000)	207.3 (183.7 to 215.5)	200.2 (192.5 to 205.4)	184.1 (177.5 to 189)	172.4 (154.6 to 188)	-16.8 (-23.4 to -0.3)
		YLLs	All ages (number)	1147522 (1028502 to 1190571)	1209395 (1163467 to 1236501)	1238729 (1186807 to 1273802)	1275334 (1147190 to 1384512)	11.1 (2.7 to 30.8)
			Age-standardized rate (per 100,000)	201.4 (178.2 to 209.4)	194.1 (186.6 to 198.5)	178.3 (172 to 182.8)	166.8 (149.4 to 181.5)	-17.2 (-23.7 to -0.8)
		YLDs	All ages (number)	33071 (23927 to 42308)	36916 (26778 to 47096)	39378 (28720 to 50104)	41256 (29588 to 54003)	24.8 (10.6 to 47.6)
			Age-standardized rate (per 100,000)	5.9 (4.3 to 7.6)	6 (4.4 to 7.7)	5.8 (4.2 to 7.4)	5.6 (4 to 7.3)	-5.3 (-15.9 to 12.2)
	Region of the Americas	Incidence	All ages (number)	31535 (29515 to 32547)	39983 (38238 to 40956)	46337 (44143 to 48085)	54660 (48037 to 62718)	73.3 (51.4 to 102)
			Age-standardized rate (per 100,000)	9.4 (8.8 to 9.7)	9.5 (9.1 to 9.8)	8.7 (8.3 to 9.1)	8.4 (7.4 to 9.7)	-10.5 (-21.8 to 4.4)
		Prevalence	All ages (number)	126194 (119385 to 129880)	163134 (158898 to 167037)	188745 (181733 to 194994)	218146 (190507 to 251815)	72.9 (50.1 to 102.8)
			Age-standardized rate (per 100,000)	37.9 (35.8 to 39)	39.3 (38.3 to 40.2)	36.4 (35 to 37.6)	35.5 (30.9 to 40.9)	-6.4 (-18.7 to 9.9)

Location type	Location name	Measure	Age, Metric	Burden				% Change (1990 to 2019)
				1990	2000	2010	2019	
		Deaths	All ages (number)	21562 (19964 to 22359)	27006 (25418 to 27826)	31624 (29509 to 32969)	37960 (34793 to 41354)	76.1 (62.6 to 100.2)
			Age-standardized rate (per 100,000)	6.4 (6 to 6.6)	6.4 (6 to 6.6)	5.8 (5.5 to 6)	5.5 (5.1 to 6)	-13.5 (-20.3 to -1.8)
		DALYs	All ages (number)	557332 (525694 to 574889)	684573 (659403 to 701494)	799032 (764464 to 827939)	941223 (872363 to 1025609)	68.9 (55 to 91.6)
			Age-standardized rate (per 100,000)	169.1 (159.9 to 174.5)	166.1 (160.2 to 170.3)	151.5 (145.2 to 156.9)	145.3 (135 to 158.5)	-14.1 (-21.3 to -2.9)
		YLLs	All ages (number)	541063 (510569 to 557893)	663701 (639848 to 680275)	775013 (741819 to 804072)	913110 (848894 to 994433)	68.8 (55 to 91.5)
			Age-standardized rate (per 100,000)	164.2 (155 to 169.3)	161.1 (155.4 to 165)	146.9 (141 to 152.4)	140.9 (130.8 to 153.6)	-14.2 (-21.4 to -3)
		YLDs	All ages (number)	16269 (11901 to 20638)	20873 (15310 to 26818)	24019 (17528 to 30740)	28113 (20462 to 37045)	72.8 (49.8 to 105.9)
			Age-standardized rate (per 100,000)	4.9 (3.6 to 6.2)	5 (3.7 to 6.4)	4.6 (3.3 to 5.8)	4.4 (3.2 to 5.8)	-10.1 (-22.2 to 7.3)
	South-East Asia Region	Incidence	All ages (number)	13631 (10037 to 21482)	21967 (17469 to 30752)	33921 (28533 to 42976)	53078 (42733 to 66649)	289.4 (139.4 to 426.4)
			Age-standardized rate (per 100,000)	3.3 (2.5 to 5.1)	4 (3.3 to 5.6)	4.6 (3.9 to 5.8)	5.6 (4.5 to 7)	68.4 (7.1 to 123.6)
		Prevalence	All ages (number)	52861 (37453 to 85556)	89395 (69462 to 126574)	138797 (114572 to 179119)	214719 (171554 to 272012)	306.2 (138.6 to 460.9)
			Age-standardized rate (per 100,000)	11.3 (8.3 to 17.7)	14.7 (11.6 to 20.5)	17.4 (14.5 to 22.3)	21.6 (17.3 to 27.3)	92 (16.1 to 159.8)
		Deaths	All ages (number)	9347 (7017 to 14370)	14496 (11834 to 20139)	22445 (19001 to 27685)	35637 (28489 to 44890)	281.3 (144.1 to 407.3)
			Age-standardized rate (per 100,000)	2.6 (2 to 3.8)	3 (2.5 to 4.1)	3.3 (2.8 to 4.1)	3.9 (3.2 to 4.9)	53.7 (0.8 to 102.3)
		DALYs	All ages (number)	314061 (229448 to 501563)	476659 (388052 to 674634)	709865 (596376 to 894702)	1076925 (857775 to 1360596)	242.9 (115.3 to 363.6)
			Age-standardized rate (per 100,000)	74.2 (55.3 to 114.8)	86.2 (70.4 to 120.6)	95.5 (80.4 to 119)	112.2 (89.6 to 141.6)	51.2 (-3.1 to 100.7)
		YLLs	All ages (number)	307278 (223874 to 492923)	465620 (378730 to 661029)	692666 (581196 to 873289)	1050130 (835674 to 1327693)	241.8 (114.5 to 361)
			Age-standardized rate (per 100,000)	72.6 (54 to 112.7)	84.2 (69.1 to 118.2)	93.2 (78.3 to 116.3)	109.4 (87 to 138.7)	50.7 (-3.5 to 100.6)
		YLDs	All ages (number)	6782 (4343 to 11067)	11039 (7507 to 16511)	17199 (11928 to 23537)	26795 (18174 to 37097)	295.1 (138.8 to 437.1)
			Age-standardized rate (per 100,000)	1.6 (1 to 2.5)	2 (1.3 to 2.9)	2.3 (1.6 to 3.1)	2.8 (1.9 to 3.8)	72.6 (8.3 to 131.2)

Location type	Location name	Measure	Age, Metric	Burden				% Change (1990 to 2019)
				1990	2000	2010	2019	
	**Western Pacific Region**	**Incidence**	All ages (number)	23884 (20823 to 29467)	36578 (33110 to 43161)	52587 (47205 to 59618)	71560 (56926 to 84580)	199.6 (117.7 to 271.5)
			Age-standardized rate (per 100,000)	3.6 (3.1 to 4.5)	4.3 (3.9 to 5.1)	4.8 (4.3 to 5.5)	5.4 (4.3 to 6.3)	49.6 (9.2 to 84.7)
		**Prevalence**	All ages (number)	102741 (88695 to 125136)	165131 (145338 to 189683)	240132 (215709 to 273249)	316474 (253975 to 374030)	208 (127 to 279.9)
			Age-standardized rate (per 100,000)	14.6 (12.8 to 17.8)	18.7 (16.6 to 21.4)	22 (19.7 to 25)	25 (20.1 to 29.5)	71 (26 to 109.9)
		**Deaths**	All ages (number)	15051 (12989 to 19307)	21999 (19973 to 26341)	31538 (27800 to 35615)	44592 (35091 to 52688)	196.3 (108.7 to 274.4)
			Age-standardized rate (per 100,000)	2.4 (2.1 to 3.1)	2.7 (2.4 to 3.2)	2.9 (2.5 to 3.3)	3.2 (2.5 to 3.7)	31.1 (-8.2 to 65.7)
		**DALYs**	All ages (number)	486108 (415073 to 610806)	692099 (628230 to 822190)	933763 (825943 to 1056185)	1247835 (992129 to 1478321)	156.7 (81.9 to 225.5)
			Age-standardized rate (per 100,000)	72.6 (62.3 to 92)	80.6 (73.4 to 95.8)	84.6 (75 to 95.5)	91.7 (73.4 to 108.8)	26.2 (-10.5 to 58.8)
		**YLLs**	All ages (number)	473515 (404055 to 596240)	672144 (609110 to 796832)	904095 (803232 to 1021609)	1207944 (957189 to 1437491)	155.1 (79.3 to 225.6)
			Age-standardized rate (per 100,000)	70.8 (60.5 to 89.8)	78.3 (71.1 to 93.1)	81.9 (72.8 to 92.5)	88.6 (70.7 to 105.8)	25.3 (-11.5 to 59.2)
		**YLDs**	All ages (number)	12593 (8769 to 17178)	19955 (14027 to 26348)	29668 (20859 to 39342)	39892 (27454 to 54420)	216.8 (129.8 to 296.6)
			Age-standardized rate (per 100,000)	1.9 (1.3 to 2.6)	2.3 (1.6 to 3.1)	2.7 (1.9 to 3.6)	3 (2.1 to 4.1)	59.9 (16.7 to 98.3)

Data in parentheses are 95% Uncertainty Intervals (95% UIs); DALYs = Disability-Adjusted Life Years; YLLs = Years of Life Lost; YLDs = Years Lived with Disability
