# Supplementary material for: Global, regional, and national quality of care index of cervical and ovarian cancer: a systematic analysis for the global burden of disease study 1990–2019
Source: BMC Womens Health. 2024 Jan 25;24:69. doi: 10.1186/s12905-024-02884-9 (PMC10809627; doi:10.1186/s12905-024-02884-9)
Supplement: Supplementary file 5 — Additional file 5: Supplementary Table 5. The QCI for ovarian cancer from 1990 to 2019 in different locations. [file 12905_2024_2884_MOESM5_ESM.pdf]

Location type	Location name	QCI				% Change (1990 to 2019)
		1990	2000	2010	2019	
Global		48.5	53.2	56.3	58.4	20.5
World Bank Income Levels	World Bank High Income	62.7	68.7	72.0	72.7	16.0
	World Bank Upper Middle Income	44.2	51.2	58.2	63.8	44.5
	World Bank Lower Middle Income	32.8	37.5	42.4	46.3	41.2
	World Bank Low Income	20.4	24.0	26.5	29.9	46.2
SDI	High SDI	62.3	68.8	72.5	73.1	17.4
	High-middle SDI	51.7	55.1	60.6	65.3	26.4
	Middle SDI	41.7	50.2	55.7	60.5	45.0
	Low-middle SDI	25.1	31.5	36.5	41.3	64.4
	Low SDI	14.6	18.3	23.4	28.2	92.9
WHO Regions	African Region	19.4	22.3	24.3	28.9	49.2
	Eastern Mediterranean Region	25.6	31.6	37.7	44.3	73.0
	European Region	58.4	61.8	64.9	67.1	14.9
	Region of the Americas	55.9	59.2	60.0	61.2	9.5
	South-East Asia Region	30.0	38.1	43.2	46.8	56.0
	Western Pacific Region	52.6	62.6	69.6	72.6	38.0
Country	Afghanistan	15.0	17.0	21.7	26.3	75.6
	Albania	58.2	65.1	69.9	73.9	27.0
	Algeria	39.9	48.3	51.9	58.5	46.6
	American Samoa	48.1	58.3	59.6	60.9	26.8
	Andorra	64.7	73.7	75.8	75.2	16.2
	Angola	11.1	14.5	20.5	25.5	130.4
	Antigua and Barbuda	50.7	47.5	50.6	50.4	-0.7
	Argentina	38.5	47.4	51.5	56.3	46.3
	Armenia	45.8	46.7	50.7	54.0	17.8
	Australia	59.9	66.2	68.1	68.2	13.8
	Austria	65.1	71.3	71.5	72.9	12.1
	Azerbaijan	50.8	50.4	52.9	57.9	13.9
	Bahamas	49.9	55.9	58.2	59.3	18.8
	Bahrain	28.4	38.0	45.7	52.3	84.1
	Bangladesh	16.3	22.8	31.5	39.2	140.2
	Barbados	51.4	55.8	57.9	58.9	14.6
	Belarus	34.3	38.9	45.7	53.7	56.2
	Belgium	57.5	62.5	65.3	66.0	14.9
	Belize	45.2	46.8	50.9	52.5	16.1
	Benin	19.3	23.5	26.3	27.5	42.6
	Bermuda	48.2	58.5	67.5	70.9	47.0
	Bhutan	13.7	22.8	32.4	39.0	184.2
	Bolivia (Plurinational State of)	19.8	28.2	35.2	40.9	106.9
	Bosnia and Herzegovina	49.8	56.4	57.7	60.2	20.9
	Botswana	26.2	31.5	34.7	43.0	64.1
	Brazil	38.3	45.6	51.6	55.8	45.6
	Brunei Darussalam	40.4	52.1	61.2	64.6	59.8
	Bulgaria	60.1	58.9	59.3	64.2	6.8
	Burkina Faso	18.4	22.8	26.9	28.1	52.8
	Burundi	10.9	12.6	19.8	21.1	93.7
	Cabo Verde	43.6	44.6	51.7	55.5	27.4
	Cambodia	32.7	42.3	52.6	60.4	84.7

Location type	Location name	QCI				% Change (1990 to 2019)
		1990	2000	2010	2019	
	Cameroon	20.1	20.1	24.0	30.4	51.5
	Canada	61.5	67.3	69.5	70.6	14.8
	Central African Republic	9.7	9.2	11.0	12.3	27.4
	Chad	16.2	15.7	18.0	21.0	29.5
	Chile	42.6	52.0	60.4	64.4	51.3
	China	46.3	56.2	63.5	67.7	46.3
	Colombia	39.6	50.2	59.9	66.5	68.0
	Comoros	11.8	17.2	18.6	26.9	127.5
	Congo	13.8	14.4	21.4	25.4	83.5
	Cook Islands	63.2	73.1	74.8	76.3	20.9
	Costa Rica	51.1	53.0	59.6	64.0	25.2
	Croatia	78.8	83.6	84.3	87.0	10.5
	Cuba	60.3	62.4	64.4	68.6	13.9
	Cyprus	47.3	57.9	68.0	67.4	42.5
	Czechia	61.0	69.8	70.9	73.1	19.8
	Côte d'Ivoire	20.9	21.1	23.9	26.9	28.7
	Democratic People's Republic of Korea	66.4	66.9	71.3	74.0	11.4
	Democratic Republic of the Congo	14.5	15.9	16.7	22.1	51.9
	Denmark	40.9	48.6	57.8	58.6	43.3
	Djibouti	15.4	15.1	20.7	26.9	74.5
	Dominica	53.0	56.8	53.1	52.0	-1.9
	Dominican Republic	37.7	49.1	50.4	54.8	45.3
	Ecuador	35.3	47.8	51.2	56.1	58.9
	Egypt	34.9	42.1	46.1	54.1	55.1
	El Salvador	37.9	50.7	56.3	60.1	58.7
	Equatorial Guinea	8.6	17.7	27.8	33.0	283.1
	Eritrea	6.4	10.6	13.7	19.2	200.5
	Estonia	44.7	48.2	55.6	58.4	30.5
	Eswatini	20.1	19.2	19.0	28.1	39.7
	Ethiopia	7.0	9.9	16.6	24.1	243.0
	Fiji	44.9	50.9	53.4	57.3	27.6
	Finland	67.9	77.7	79.9	79.5	17.2
	France	59.2	63.8	67.6	69.0	16.6
	Gabon	19.0	21.2	26.2	32.4	70.5
	Gambia	18.9	21.5	22.3	26.3	39.6
	Georgia	57.2	60.8	52.1	51.9	-9.3
	Germany	68.1	74.4	75.3	75.9	11.4
	Ghana	26.5	29.5	31.8	36.5	38.1
	Greece	65.7	67.2	73.4	73.3	11.6
	Greenland	32.7	39.3	44.8	49.3	50.7
	Grenada	44.0	51.4	50.5	52.4	19.1
	Guam	61.9	74.2	74.3	72.7	17.5
	Guatemala	32.5	40.8	47.4	48.0	47.8
	Guinea	13.2	15.2	18.7	21.7	65.0
	Guinea-Bissau	17.6	19.6	22.5	25.3	44.2
	Guyana	33.6	41.3	42.0	47.5	41.6
	Haiti	19.3	23.0	21.5	28.6	48.4
	Honduras	28.2	33.0	36.0	39.1	39.0

Location type	Location name	QCI				% Change (1990 to 2019)
		1990	2000	2010	2019	
	Hungary	55.2	59.6	59.2	62.8	13.8
	Iceland	65.7	72.2	76.5	78.2	19.0
	India	20.3	25.4	31.3	36.0	77.3
	Indonesia	42.0	51.4	57.4	61.9	47.4
	Iran (Islamic Republic of)	46.4	56.9	58.1	62.8	35.2
	Iraq	38.9	43.4	53.1	62.0	59.1
	Ireland	58.9	65.8	67.9	71.1	20.8
	Israel	51.3	58.6	63.2	66.0	28.5
	Italy	69.1	74.0	76.3	77.6	12.3
	Jamaica	46.8	51.3	56.2	55.7	18.9
	Japan	68.5	82.8	91.7	91.4	33.4
	Jordan	35.4	42.0	52.9	59.4	67.6
	Kazakhstan	50.3	47.8	50.0	58.4	16.2
	Kenya	21.6	25.1	11.6	20.0	-7.2
	Kiribati	35.0	42.8	45.4	46.4	32.6
	Kuwait	51.4	58.9	57.1	64.8	26.1
	Kyrgyzstan	42.3	45.1	46.9	51.1	20.7
	Lao People's Democratic Republic	27.5	36.7	47.0	54.3	97.3
	Latvia	25.0	26.2	29.6	33.7	34.8
	Lebanon	39.8	50.6	58.5	64.9	63.0
	Lesotho	17.6	14.5	13.7	20.0	14.0
	Liberia	16.8	20.8	25.0	26.4	57.1
	Libya	35.9	41.9	50.2	49.3	37.4
	Lithuania	32.4	36.6	40.5	42.6	31.4
	Luxembourg	57.9	67.9	70.3	67.8	17.1
	Madagascar	18.3	19.2	21.2	23.6	29.0
	Malawi	17.2	17.7	21.5	25.3	47.0
	Malaysia	46.4	59.0	66.8	72.3	55.7
	Maldives	40.0	56.2	73.4	79.1	97.6
	Mali	16.6	22.1	25.8	28.8	73.7
	Malta	55.0	62.9	68.1	72.3	31.4
	Marshall Islands	37.6	41.0	44.5	47.9	27.3
	Mauritania	17.3	21.7	24.9	31.5	82.1
	Mauritius	62.2	72.9	76.5	79.5	27.9
	Mexico	39.8	51.2	55.9	59.3	49.2
	Micronesia (Federated States of)	37.2	44.4	49.4	53.6	44.2
	Monaco	67.4	70.7	73.2	74.2	10.2
	Mongolia	32.6	31.0	38.1	43.4	33.3
	Montenegro	64.9	64.6	64.7	67.9	4.5
	Morocco	25.6	33.8	39.4	47.0	83.7
	Mozambique	9.5	14.7	17.3	22.2	133.2
	Myanmar	33.2	42.7	49.6	59.4	78.8
	Namibia	18.2	20.0	25.8	34.6	90.4
	Nauru	50.3	52.8	53.1	61.8	23.0
	Nepal	15.1	22.3	27.2	32.9	117.5
	Netherlands	63.0	66.1	68.5	69.3	10.0
	New Zealand	61.1	63.6	64.8	66.2	8.3
	Nicaragua	41.6	43.3	50.5	56.4	35.6

Location type	Location name	QCI				% Change (1990 to 2019)
		1990	2000	2010	2019	
	Niger	15.2	17.9	21.6	24.0	58.2
	Nigeria	18.5	22.1	25.9	27.3	48.0
	Niue	55.2	65.3	70.9	72.4	31.1
	North Macedonia	51.9	57.0	58.8	61.6	18.7
	Northern Mariana Islands	62.6	75.7	73.8	73.3	17.2
	Norway	61.8	68.7	71.3	69.5	12.5
	Oman	36.6	49.5	52.9	57.8	57.9
	Pakistan	18.3	21.0	27.0	33.6	83.6
	Palau	53.2	62.8	65.4	67.8	27.5
	Palestine	39.5	44.4	44.3	51.6	30.6
	Panama	45.5	51.9	56.0	62.9	38.3
	Papua New Guinea	32.4	39.6	40.6	43.5	34.4
	Paraguay	42.5	45.5	47.0	54.1	27.1
	Peru	35.3	45.3	53.7	61.6	74.7
	Philippines	51.8	60.6	64.1	67.5	30.3
	Poland	40.7	47.2	47.8	49.5	21.6
	Portugal	51.2	54.5	57.7	59.5	16.3
	Puerto Rico	54.8	59.1	65.3	68.4	24.8
	Qatar	29.7	37.0	45.0	52.4	76.3
	Republic of Korea	52.5	68.3	83.6	86.1	64.0
	Republic of Moldova	37.9	41.9	42.4	47.2	24.6
	Romania	58.8	61.8	61.3	64.8	10.2
	Russian Federation	53.1	51.3	57.5	62.2	17.2
	Rwanda	10.4	10.9	21.1	28.0	170.2
	Saint Kitts and Nevis	48.8	53.4	50.8	50.6	3.5
	Saint Lucia	42.3	49.0	59.6	56.5	33.7
	Saint Vincent and the Grenadines	45.1	50.8	52.4	51.6	14.4
	Samoa	44.4	54.3	56.6	58.6	31.9
	San Marino	63.4	69.4	73.2	76.2	20.2
	Sao Tome and Principe	20.3	26.3	32.3	38.1	87.8
	Saudi Arabia	16.2	24.5	53.9	64.8	301.1
	Senegal	20.3	22.6	23.8	27.2	34.3
	Serbia	44.2	49.4	53.1	55.6	25.8
	Seychelles	57.3	64.3	71.7	75.0	31.0
	Sierra Leone	18.4	19.7	21.7	26.3	42.9
	Singapore	54.8	67.5	78.9	83.0	51.4
	Slovakia	65.2	72.6	77.4	81.0	24.2
	Slovenia	40.9	44.6	48.1	52.0	27.2
	Solomon Islands	43.6	53.0	54.1	56.9	30.5
	Somalia	8.8	8.4	7.8	10.1	15.7
	South Africa	32.0	28.7	29.3	35.6	11.3
	South Sudan	11.2	11.7	14.0	15.4	37.3
	Spain	89.8	90.4	92.0	92.0	2.5
	Sri Lanka	57.3	64.3	72.2	79.6	39.0
	Sudan	24.3	32.2	37.4	45.2	86.1
	Suriname	37.9	40.6	45.8	48.8	28.8
	Sweden	63.0	63.5	59.8	62.3	-1.2
	Switzerland	70.6	75.3	72.4	72.4	2.6

Location type	Location name	QCI				% Change (1990 to 2019)
		1990	2000	2010	2019	
	Syrian Arab Republic	41.2	52.8	58.4	60.4	46.7
	Taiwan (Province of China)	76.3	88.4	98.3	100.0	31.1
	Tajikistan	32.4	31.7	30.5	34.0	4.9
	Thailand	52.9	70.4	78.0	82.5	56.0
	Timor-Leste	29.8	40.1	47.6	55.9	87.5
	Togo	23.5	22.7	25.6	30.2	28.6
	Tokelau	44.7	53.8	60.4	64.8	45.2
	Tonga	43.7	51.7	53.2	56.8	29.8
	Trinidad and Tobago	42.5	45.6	51.5	54.6	28.3
	Tunisia	39.6	48.5	55.7	61.5	55.3
	Turkey	30.4	43.6	54.4	59.7	96.2
	Turkmenistan	53.8	58.2	61.5	65.8	22.2
	Tuvalu	40.3	49.9	52.8	56.4	40.1
	Uganda	12.1	16.8	21.2	27.2	124.2
	Ukraine	59.6	59.7	67.9	71.5	19.9
	United Arab Emirates	36.9	43.3	41.7	53.4	44.6
	United Kingdom	60.0	67.2	74.0	75.0	25.0
	United Republic of Tanzania	16.9	19.5	23.7	27.7	63.7
	United States Virgin Islands	42.5	49.0	52.9	52.2	22.7
	United States of America	65.5	67.5	66.0	65.1	-0.6
	Uruguay	44.6	50.8	55.6	57.3	28.6
	Uzbekistan	48.9	48.9	44.1	48.7	-0.4
	Vanuatu	35.0	44.7	44.2	45.7	30.7
	Venezuela (Bolivarian Republic of)	46.3	53.4	60.4	63.7	37.6
	Viet Nam	40.1	51.7	62.4	69.1	72.1
	Yemen	22.2	27.8	34.4	37.1	67.4
	Zambia	14.1	14.6	21.6	30.6	117.1
	Zimbabwe	28.1	44.9	25.5	28.0	-0.3
