# Supplementary material for: Global, regional, and national quality of care index of cervical and ovarian cancer: a systematic analysis for the global burden of disease study 1990–2019
Source: BMC Womens Health. 2024 Jan 25;24:69. doi: 10.1186/s12905-024-02884-9 (PMC10809627; doi:10.1186/s12905-024-02884-9)
Supplement: Supplementary file 6 — Additional file 6: Supplementary Table 6. The QCI for ovarian cancer from 1990 to 2019 among different age groups. [file 12905_2024_2884_MOESM6_ESM.pdf]

Age group	Year			
	1990	2000	2010	2019
5-14 years	53.8	59.7	63.7	66.1
5 to 9	52.5	58.9	63.2	65.9
10 to 14	54.4	60.1	63.8	66
15-49 years	59	62.5	66.9	68.2
15 to 19	59.9	65.4	69.5	70.8
20 to 24	60.9	66	70.8	71.5
25 to 29	61.7	65.5	70.3	71.8
30 to 34	60.5	64	68.5	70.5
35 to 39	60.9	65.1	69.2	70.6
40 to 44	61.3	65.4	69.5	70.3
45 to 49	57	61.2	65.9	66.9
50-69 years	52.2	57.1	60.3	60
50 to 54	52	58.2	61.1	62.1
55 to 59	52.4	58	60.1	61.1
60 to 64	52.9	56.7	59.7	59.4
65 to 69	54.1	56.3	58.4	58
70+ years	50.9	53.2	52.5	52.6
70 to 74	51.2	53	53.3	54.2
75 to 79	48.9	52.6	51.3	51.6
80+ years	51.1	51.9	52.1	51.1
Age-standardized	48.5	53.2	56.3	58.4
